# *Slc20a1* and *Slc20a2* regulate neuronal plasticity and cognition independently of their phosphate transport ability

**DOI:** 10.1038/s41419-023-06292-z

**Published:** 2024-01-09

**Authors:** Mariana Ramos-Brossier, David Romeo-Guitart, Fabien Lanté, Valérie Boitez, François Mailliet, Soham Saha, Manon Rivagorda, Eleni Siopi, Ivan Nemazanyy, Christine Leroy, Stéphanie Moriceau, Sarah Beck-Cormier, Patrice Codogno, Alain Buisson, Laurent Beck, Gérard Friedlander, Franck Oury

**Affiliations:** 1 Université Paris Cité, INSERM UMR-S1151, CNRS UMR-S8253, Institut Necker Enfants Malades, Team 8, F-75015 Paris, France; 2 Univ. Grenoble Alpes, Inserm, U1216, Grenoble Institut Neurosciences, 38000 Grenoble, France; 3 Institut Pasteur, Perception & Memory Unit, F-75015 Paris, France; 4 MedInsights, 6 rue de l’église, F-02810 Veuilly la Poterie, France; 5 Platform for Metabolic Analyses, Structure Fédérative de Recherche Necker, INSERM US24/CNRS UAR, 3633 Paris, France; 6 Université Paris Cité, INSERM UMR-S1151, CNRS UMR-S8253, Institut Necker Enfants Malades, Team 6, F-75015 Paris, France; 7 Platform for Neurobehavioural and metabolism, Structure Fédérative de Recherche Necker, INSERM, US24/CNRS UAR, 3633 Paris, France; 8 Institute of Genetic Diseases, Imagine, 75015 Paris, France; 9 Nantes Université, CNRS, Inserm, l’Institut du Thorax, F-44000 Nantes, France

**Keywords:** Molecular neuroscience, Hippocampus, Protein transport, Transporters in the nervous system, Long-term depression

## Abstract

In recent years, primary familial brain calcification (PFBC), a rare neurological disease characterized by a wide spectrum of cognitive disorders, has been associated to mutations in the sodium (Na)-Phosphate (Pi) co-transporter *SLC20A2*. However, the functional roles of the Na-Pi co-transporters in the brain remain still largely elusive. Here we show that *Slc20a1* (PiT-1) and *Slc20a2* (PiT-2) are the most abundant Na-Pi co-transporters expressed in the brain and are involved in the control of hippocampal-dependent learning and memory. We reveal that *Slc20a1* and *Slc20a2* are differentially distributed in the hippocampus and associated with independent gene clusters, suggesting that they influence cognition by different mechanisms. Accordingly, using a combination of molecular, electrophysiological and behavioral analyses, we show that while PiT-2 favors hippocampal neuronal branching and survival, PiT-1 promotes synaptic plasticity. The latter relies on a likely Otoferlin-dependent regulation of synaptic vesicle trafficking, which impacts the GABAergic system. These results provide the first demonstration that Na-Pi co-transporters play key albeit distinct roles in the hippocampus pertaining to the control of neuronal plasticity and cognition. These findings could provide the foundation for the development of novel effective therapies for PFBC and cognitive disorders.

## Introduction

Solute carrier proteins (SLC) represent the largest family of transmembrane transporters in mammalian cells [1–3]. They are essential for the maintenance of whole-body homeostasis by mediating the transport of solutes across cell membranes via ionic gradients, functioning as exchangers or facilitating the passive diffusion of specific solute molecules [4–8]. In the brain, SLCs are highly expressed in various regions, such as the hippocampus (HpC) [9, 10]. Mounting evidence suggests that dysregulations of various SLCs is associated with the development of numerous brain disorders such as neurodegenerative diseases, epilepsy or autism [11–14].

Recent work has shown that primary familial brain calcification (PFBC), an inherited form of neurological disorder with a wide spectrum of cognitive aspects, is associated to mutations in *SLC20A2*, *a* sodium (Na)-Phosphate (Pi) co-transporter [15–18]. Nevertheless, the etiology of brain calcification in PFBC patients and the involvement of Na-Pi co-transporter in the development of this particular neurological disease are still unclear.

Na-Pi cotransporters are composed by 3 families of SLCs, SLC17, SLC34 and SLC20. These families differ largely in their amino acid sequence and structure, Pi transport characteristics, tissue distribution and physiological roles [19, 20]. SLC20 Na-Pi co-transporters, including *SLC20A1* (PiT-1) and *SLC20A2 (*PiT-2), are highly expressed in the central nervous system, while SLC17 and SLC34 families are almost undetectable [21–23]. Both PiT-1 and PiT-2 were initially identified as cell surface receptors for Gibbon Ape Leukemia (Glvr1) and amphotrophic (Ram1) retroviruses respectively [24, 25], and were later found to transport Pi when expressed in mammalian cells [26–28]. Human PiT-1 and PiT-2 share 62% identity at the amino acid level, and 2D protein prediction topology models predict 12 trans-membrane spanning domains with a singular large intracellular domain [29–31]. The broad tissue distribution of PiT-1 and PiT-2 first led to the assumption that they played house-keeping roles in supplying Pi to cells [27, 32]. However, significant differences in their cellular expression levels and ratios within tissues challenged this view and suggested cell-specific non-redundant functions. Accordingly, numerous additional roles for PiT-1 were identified, independent of its Pi transport activity, including cell proliferation [29, 33, 34], chondrocyte survival [35], embryonic mouse liver development [29], tumor necrosis factor (TNF)-induced apoptosis [30], erythroid and B cell differentiation [36, 37], control of energy metabolism, insulin signaling [38] and inflammation [39]. *SLC20A1* mutations in humans were recently discovered in patients with bladder exstrophy-epispadias complex (BEEC), a disease that is not characterized by impairments in Pi transport, pointing to non-canonical, transport-independent roles for Na-Pi transporters [40, 41]. However, the exact role and functional relevance of brain-borne Na-Pi co-transporters are still unknown.

In this study, we set out to explore the physiological roles of PiT-1 and PiT-2 in brain functions and neuronal activity. By using a combination of large-scale transcriptomics, molecular, histological, electrophysiological and behavioral analyses, we provide evidence that both proteins are highly expressed in the hippocampus and affect hippocampal-dependent functions such as memory and that each regulate different aspects of neuronal homeostasis and plasticity independently of Pi transport.

## Results

### *Slc20a1* and *Slc20a2* are the only Na-Pi co-transporters expressed in the hippocampus with differential spatial distributions

**Fig. 1:**
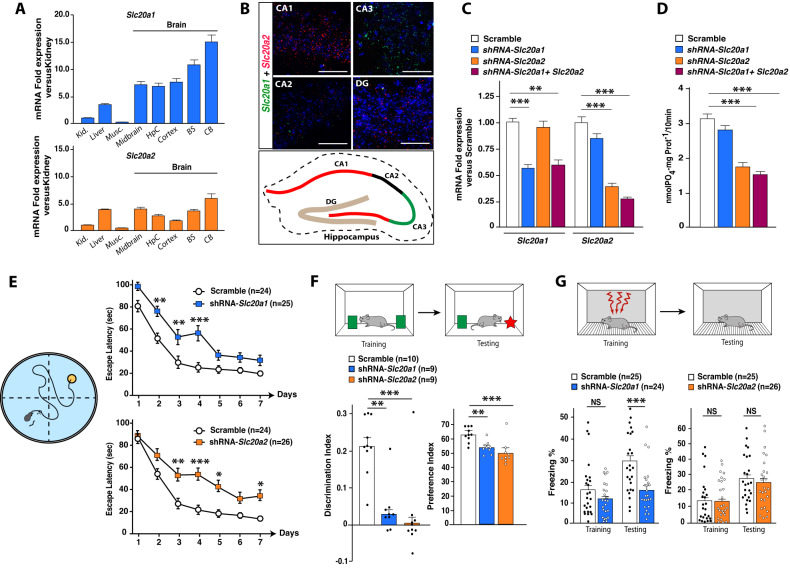
Hippocampal downregulation of either *Slc20a1* or *Slc20a2* induces learning and memory deficits. **A** Relative expression of the *Slc20a1* and *Slc20a2* genes in kidney, liver, muscle, and various parts of the brain (Midbrain, Hippocampus (HpC), Cortex, Brainstem (BS), and Cerebellum (CB), *n* = 3 adult mice). **B** In situ hybridization of *Slc20a1* (green) and *Slc20a2* (red) mRNAs of hippocampal brain sections (Scale bar = 200 μm). A focus is made on the CA1, CA2, CA3 and Dentate Gyrus (DG) areas. The analysis was performed on *n* = 3 adult mice **C** *Slc20a1* and *Slc20a2* mRNA expression (qPCR performed in triplicate for each sample) in primary hippocampal neurons transduced with lentiviruses expressing either shRNA-*Slc20a1*, shRNA-*Slc20a2*, shRNA-*Slc20a1*+shRNA-*Slc20a2* or control. These measurements were obtained from *n* = 7–12 wells from 3 independent neuronal preparations. **D** Phosphate transport analysis (nM PO4 x mg prot-1/10 min) performed on primary hippocampal neurons transduced with the same lentiviruses as above. Experiments were performed in 3 independent cultures, and measurements were assessed in triplicate (3 wells in one plate). Values reported are the mean value of each plate (*n* = 5–9). **E** Morris Water Maze (MWM) was performed in mice 3 weeks after hippocampal stereotactic injections (AAV) with either Scramble, shRNA-*Slc20a1* or shRNA-*Slc20a2*. The graph shows the mean time (in seconds, over four trials per day for 7 consecutive days) needed for each group of mice to localize the submerged platform in the swimming area. **F** Novel object recognition (NOR) was performed in 3 groups of mice: Scramble, shRNA-*Slc20a1* or shRNA-*Slc20a2*. Discrimination index was measured 24 hours after the training phase to assess memory performance. **G** Contextual Fear Conditioning (CFC) was performed in mice after hippocampal stereotactic injections of AAV expressing either Scramble, shRNA-*Slc20a1* or shRNA-*Slc20a2*. The percentage of freezing was measured for the training and testing phases. All behavioral tests were performed in at least two independent experiments for **E**, 1 cohort for **F**, and three independent experiments for **G.** Data are expressed as mean ± s.e.m. *p ≤ 0.05, **p ≤ 0.01, ***p ≤ 0.001, NS: not significant, by two-tailed Student’s *t*-test or 2-way ANOVA followed by Tukey’s multiple comparisons test compared to the control group.

We investigated the expression patterns of the various SLC17, 20 and 34 Na-Pi co-transporter families in the mouse brain using reverse transcription-polymerase chain reaction (RT-PCR). We confirmed that *Slc20* family members (*Slc20a1* and *Slc20a2)* are the most abundantly Na-Pi co-transporters genes expressed in the mouse brain, while *Slc17* and *Slc34* families were almost undetectable (Fig. 1A and S1A). By in situ hybridization, we found that *Slc20a1* and *Slc20a2* are highly expressed in the HpC but exhibit a differential spatial distribution in this brain area (Fig. 1B). More precisely, *Slc20a1* is strongly expressed in the CA3 region of the HpC, while *Slc20a2* is preferentially expressed in the CA1 and dentate gyrus (DG) regions. PiT-1 and PiT-2 were first described for their ability to transport Pi across the plasma membrane [25, 26]. Therefore, using primary HpC neuronal cultures after targeting *Slc20a1*, *Slc20a2*, or both by lentiviral-mediated shRNAs, we measured the contribution of both proteins in Pi transport, (Fig. 1C). The downregulation of either *Slc20a1* or *Slc20a2* induced a significant decrease in the expression of the target gene without affecting the expression of the paralogous *Slc20a* gene (Fig. 1C). Importantly, we found that Na-dependent Pi transport did not decrease following downregulation of *Slc20a1*, but decreased by 50% upon knockdown of *Slc20a2*, with no further significant decrease when targeting both genes (Fig. 1D). Furthermore, we have found that *Slc20a2* downregulation in primary neuronal cultures slightly increases the extracellular Pi concentration (0.83 mM for control and 0.88 mM after Slc20a2 downregulation) (Fig. S1B), which is consistent with the decreased in Pi transport (Fig. 1D) and the role of PiT-2 as a Pi transporter. However, this increase is very low and we could not detect any calcium-phosphate crystal formation in these conditions (Fig. S1C). This latter observation is consistent with previous studies showing that the formation of those crystals in neurons would only occur when extracellular Pi is largely increased [42–44]. Of a note, we did not observe any induction of the expression of the other Na-Pi co-transporters genes family in the HpC after local stereotaxic injections of adeno-associated viruses (AAV-9) expressing shRNAs targeting either *Slc20a1* or *Slc20a2* in the HpC of adult mice (Fig. S1D-F). These results indicate that Pi transport in these experimental conditions is partially mediated by the activity of PiT-2, and not PiT-1, and suggest that other non-defined mechanisms could be involved for Pi import in neurons [35, 45]. In addition, the differential localization and Pi transport activities suggest that PiT-1 and PiT-2 may also have different roles in the HpC, and may exert Pi transport-independent functions in HpC neurons.

### Hippocampal PiT-1 and PiT-2 influence cognitive functions

To investigate the functional relevance of PiT-1 and PiT-2, we have subjected adult mice downregulated for either *Slc20a1* or *Slc20a2* in the HpC to a series of behavioral tasks to assess cognitive functions, namely spatial learning and memory (Morris Water Maze test, MWM), episodic memory (novel object recognition test, NOR), associative memory (contextual fear conditioning, CFC), and exploratory/anxiety-like behavior (open field test (OFT) and light/dark transition test (L/DT)) (Fig. 1E-G and S1G-I). We showed that HpC downregulation of either *Slc20a1* or *Slc20a2* produced a significant delay in spatial learning as depicted by an increased latency to locate the Morris water maze platform compared to control mice (injected with AAV-shRNA-Scramble) (Fig. 1E). In addition, we found that both *Slc20a1* and *Slc20a2* downregulation in the HpC severely impaired episodic memory, illustrated by a drastic decrease in the time exploring a novel object compared to control mice (Fig. 1F and S1G). Moreover, we observed that contextual fear memory was impaired by *Slc20a1* downregulation, resulting in a decrease of context-elicited freezing time during the testing phase of this task. Interestingly, this was not seen after *Slc20a2* downregulation, as previously described in *Slc20a2* knockout mice [46] (Fig. 1G). Our results also showed that solely *Slc20a2* downregulation impacted exploratory- and anxiety-like behaviors, as demonstrated by our results in the OFT and D/LT tests (Fig. S1H-I). Taken together, these findings suggest that the differential localization of *Slc20a1* and *Slc20a2* within the HpC could be associated to distinct functional roles of PiT-1 and PiT-2 in cognition and emotional regulation.

### Downregulation of *Slc20a1* and *Slc20a2* in the hippocampus induces independent and differential gene expression pattern modifications

**Fig. 2:**
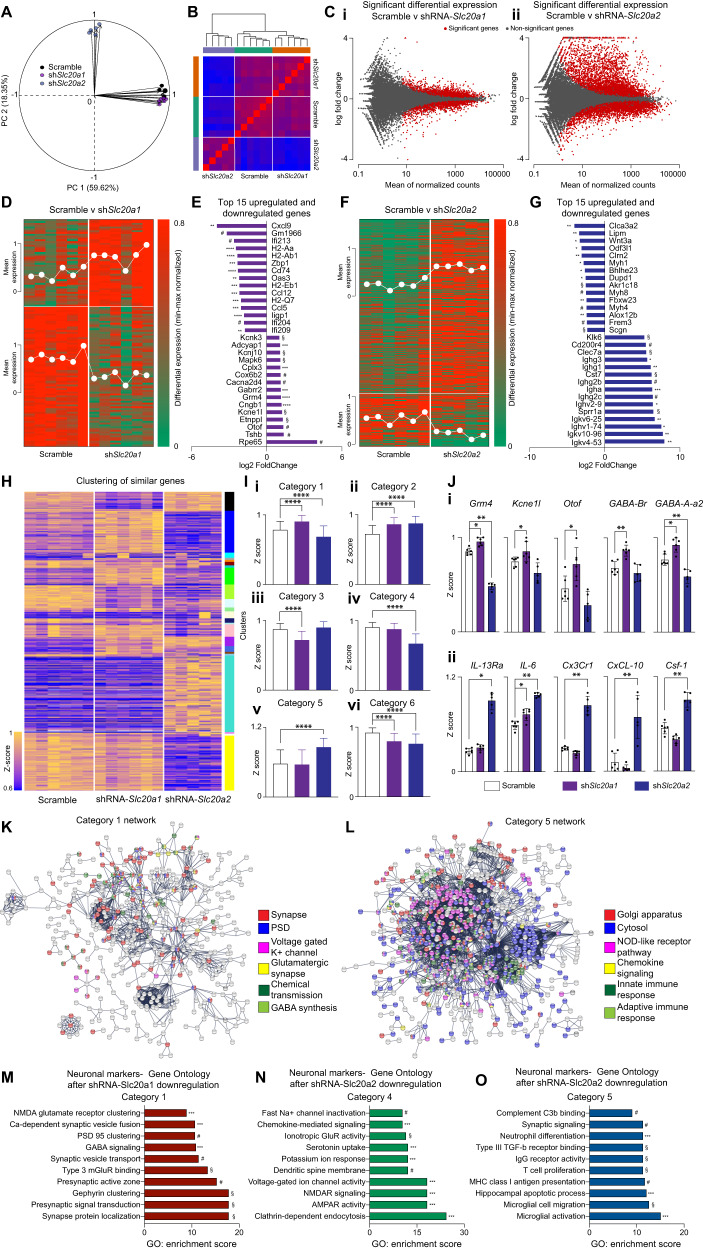
PiT-1 and PiT-2 are associated to independent gene clusters in the hippocampus. **A**–**O** RNAseq analysis performed on hippocampi after selective downregulation of either *Slc20a1* (*n* = 6) or *Slc20a2* (*n* = 5). The analyses were performed in comparison to Scramble group (*n* = 6). **A** Principal component analysis (PCA) biplot representation of the variability in the dataset along the first two PC axes (PC1-59.62% variability; PC2-18.35% variability). While shRNA-*Slc20a1* and Scramble aligns on PC1, shRNA-*Slc20a2* is orthogonal to both. **B** Heatmap obtained from sample-to-sample Euclidean distances using rlog transformation (DESeq2 pipeline) shows that shRNA-*Slc20a2* differs from both the Scramble and shRNA-*Slc20a1* groups. **C** MA plots (M-log ratio of fold change; A-mean of normalized counts) for differentially significant gene expression in shRNA-*Slc20a1* compared to Scramble (i) and for shRNA-*Slc20a2* compared to Scramble (ii). **D** Heatmap representation of the differentially downregulated and upregulated genes (min-max normalized) in Scramble vs shRNA-*Slc20a1* groups. The mean normalized expression is overlaid on the heatmap for up- and downregulated genes. Heatmap scale-0 to 0.8. **E** Bar graph representation of the top 15 up- and downregulated genes in differential expression analysis of Scramble vs shRNA-*Scl20a1*. **p < 10^-2^; ***p < 10^-3^; ****p < 10^-4^; ^#^*p* < 10^-5^–10^-10^; ^§^*p* < 10^-10^. **F** Heatmap representation of the differentially downregulated and upregulated genes (min-max normalized) in Scramble vs shRNA-*Slc20a2* groups. The mean normalized expression is overlaid on the heatmap for up- and downregulated genes. Heatmap scale-0 to 0.8. **G** Bar graph representation of the top 15 down- and upregulated genes in differential expression analysis of Scramble vs shRNA-*Slc20a2*. **p < 10^-2^; ***p < 10^-3^; ****p < 10^-4^; ^#^*p* < 10^-5^–10^-10^; ^§^*p* < 10^-10^. **H** Heatmap showing similarly varying genes clustered into 21 clusters by WCGNA analysis. The colors on the right indicate cluster identity while the heatmap is plotted using the z-score of each gene across the different groups. Heatmap scale-0.6 to 1. **I** Bar-graphs showing the mean z-score after categorization of the obtained clusters into six major categories:(i) Category 1: Upregulation in shRNA-*Slc20a1* and downregulation in shRNA-*Slc20a2*; (ii) Category 2: Upregulation in both shRNA-*Slc20a1* and shRNA-*Slc20a2*; (iii) Category 3: Downregulation in shRNA-*Slc20a1*; (iv) Category 4: Downregulation in shRNA-*Slc20a2*; (v) Category 5: Upregulation in shRNA-*Slc20a2*; (vi) Category 6: Downregulation in both shRNA-*Slc20a1* and shRNA-*Slc20a2*. Data is represented as Mean ± SD. ****p < 0.00001. **J** Mean z-score of significant genes involved in: (i) Synaptic plasticity genes (*Grm4*, *Kcne1l*, *Otof*, *GABA-Br*, *GABA-A-α2*) are upregulated in shRNA-*Slc20a1* and downregulated in shRNA-*Slc20a2*; (ii) Immune genes (*IL-13Rα*, *IL-6*, *Cx3Cr1*, *CxCL-10*, *Csf-1*) are upregulated in shRNA-*Slc20a2* group. Data is represented as Mean ± SD. *p < 0.01; **p < 0.001; ns, non-significant. **K** Gene ontology network obtained from genes in Cluster 1 showing enrichment for synaptic plasticity and its associated functions. **L** Gene ontology network obtained from genes in Cluster 5 showing enrichment for immune processes and its associated functions. **M** GO enrichment score for significant biological processes obtained from Cluster 1. ***p < 10^-3^; ^#^*p* < 10^-5^–10^-10^; §, *p* < 10^-10^. **N** GO enrichment score for significant biological processes obtained from Cluster 4. ***p < 10^-3^; ^#^*p* < 10^-5^–10^-10^; §, *p* < 10^-10^. **O** GO enrichment score for significant biological processes obtained from Cluster 5. ***p < 10^-3^; ^#^*p* < 10^-5^ –10^-10^; ^§^*p* < 10^-10^.

To explore the molecular pathways underlying the behavioral deficits elicited by PiT-1 and PiT-2 downregulation in the HpC, we performed comparative transcriptome profiling using large-scale RNA sequencing (RNA-seq) approach on HpC samples injected with AAVs expressing shRNAs targeting either *Slc20a1*, *Slc20a2* or Scramble. We used principal component analysis (PCA) as an exploratory data analysis step (Fig. 2A) through linear combinations of gene expression in our experimental groups. Analyzing PCA biplots we found that the shRNA-*Slc20a2* group was mutually orthogonal to both the Scramble and shRNA-*Slc20a1* groups (Fig. 2A), indicating that shRNA-*Slc20a2* effects were unrelated to differential gene expressions induced by shRNA-*Slc20a1* (Fig. 2A, B and S2B). The MA-plot between the two experimental groups (shRNA-*Slc20a1*- and -*Slc20a2*) and the control group (Scramble) indicated that the number of differentially expressed genes was larger in the shRNA-*Slc20a2* group compared to the shRNA-*Slc20a1* group (Fig. 2C) (red dots are differentially expressed genes at *p* = 0.01). Gene-wise differential expression analysis was performed using the DESeq2 pipeline. After estimating the gene-wise dispersion using maximum likelihood (Fig S2A; for details see Methods), differential gene expression [shRNA-*Slc20a1* vs Scramble (Fig. 2D-E); shRNA-*Slc20a2* vs Scramble (Fig. 2F, G)] was analyzed. Interestingly, downregulation of *Slc20a2* in the HpC resulted in the upregulation of major inflammation-associated genes (such as *IL13ra*, *IL-6*, *Cx3Cr1*, *CxCL-10*, *Csf-1*). Gene cluster analysis identified 3 important hubs of genes selectively linked to *Slc20a2* downregulation: neuronal homeostasis, neuroinflammation/innate immunity and neuronal apoptosis. By contrast, *Slc20a1* downregulation is associated with gene clusters involved in synaptic transmission and plasticity, with a downregulation of major genes involved in calcium (Ca^2+^)-dependent vesicular fusion and exocytosis and an upregulation of genes involved in GABAergic system function (*Gabr2A, Gabr2, Kcnj10L*). One element that stood out in our analysis was the shRNA-*Slc20a1-*induced upregulation of Otoferlin (*Otof*), which is a large multi–C2 domain protein thought to act as a calcium sensor that regulates ER-dependent synaptic vesicle trafficking and clathrin-mediated endocytosis (Fig. 2E–G). Taken together, our data support the notion that PiT-1 and PiT-2 are involved in independent functions in the HpC that could directly impact neuronal integrity, survival and plasticity.

To gain insight into the biological and molecular processes underlying these independent functions, we applied a hierarchical cluster-based approach that allowed us to assess the biological and molecular processes involved in the *Slc20a1* and *Slc20a2* pathways. To complement the differential expression analysis, we clustered the top 10% of the variable genes using a weighted correlation network analysis (WGCNA) [47]. Gene clustering was performed across the experimental groups using a topological overlay mapping with a dynamic tree cut (Fig. 2H and S2C). The categories obtained hence forth describe the global trends of gene variations (*p* < 0.0001; Fig. 2I). Consistent with our differential analyses, *GABA-Br, GABA-A-a2, Kcnj10* and *Otof* showed significant and specific upregulation in the shRNA-*Slc20a1* group (Fig. 2J-I). In contrast, *IL13-Ra*, *IL 6*, *CX3CR1*, *CXCL-10* and *CSF-1* genes showed strong upregulation in the shRNA-*Slc20a2* group (Fig. 2J -ii).

After classifying the genes into different clusters, we performed gene ontology (GO) analysis to determine the impact of expression changes induced by *Slc20a1* and *Slc20a2* downregulation. The GO and detailed enrichment analyses showed that genes specifically associated with *Slc20a1* downregulation are implicated in pre-synaptic vesicle fusion and trafficking and post-synaptic density (PSD), Ca^2+^-dependent synaptic vesicle fusion and transport, glutamatergic synapses, GABA synthesis and chemical transmission (**Category 1;** Fig. 2K and M). On the other hand, the group of genes upregulated in the shRNA-*Slc20a2* group are implicated in post-synaptic density (PSD) and synaptic signaling, chemical synapse transmission and neurotransmitter mediated postsynaptic changes (KEGG pathways) (**Category 4**; Fig. 2L and N), immune processes (NOD-like receptor pathway and chemokine signaling), and innate and adaptive immune responses (KEGG pathways) (**Category 5**; Fig. 2L and O). Significant GO enrichment of immunological processes associated to *Slc20a2* downregulation were also observed: complement C3b binding, neutrophil differentiation, Type III TGF-b receptor binding, IgG receptor activity, T cell proliferation, MHC class I antigen presentation, HpC apoptotic process, microglial cell migration and microglial activation (Fig. 2O). In summary, these analyses indicate that PiT-1 is preferentially involved in GABAergic system function, synaptic vesicle fusion and membrane trafficking, while PiT-2 is rather involved in neuronal survival and immune response control within the HpC.

### PiT-2 is required to maintain neuronal morphology and survival in the hippocampus

**Fig. 3:**
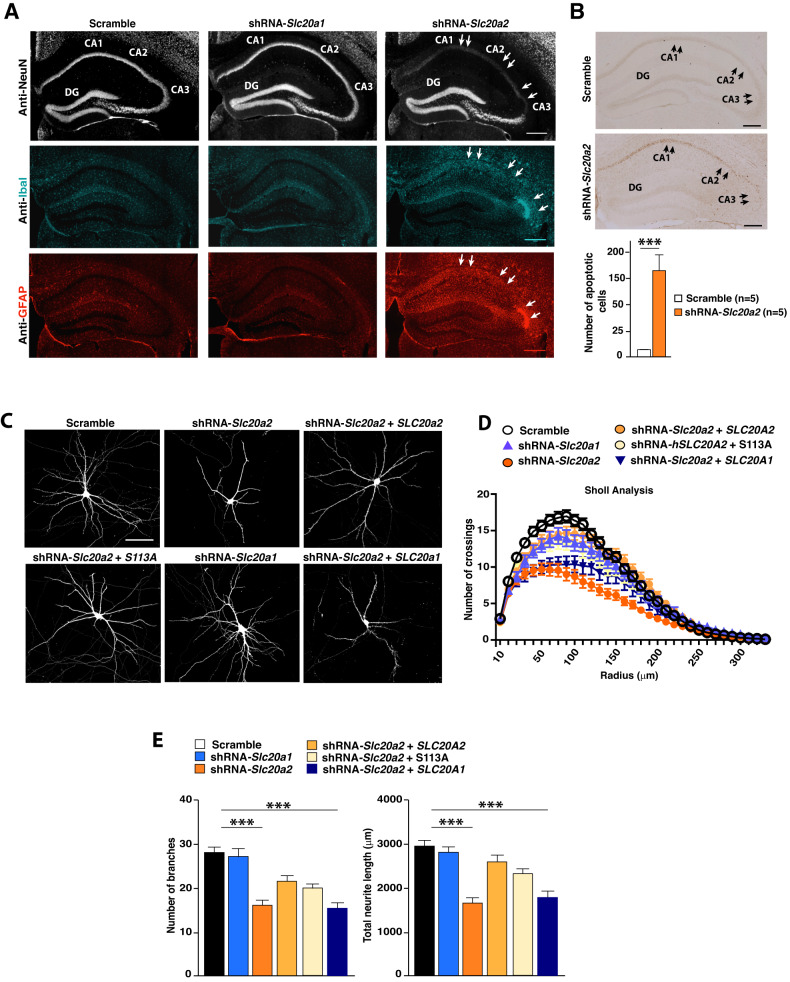
PiT-2 is required for neuronal homeostasis and survival in the hippocampus. **A** Representative images of NeuN, Iba1 and GFAP immunofluorescence performed on brain hippocampal cross-sections 3 weeks after selective stereotactic injection of either: AAV-shRNA-*Slc20a1*, shRNA-*Slc20a2* or Scramble. Arrows indicate the reduction of neurons (NeuN+ cells) and the increased of glial cells (Iba1^+^ and GFAP^+^) in the CA1, CA2 and CA3 regions of the hippocampus in shRNA-*Slc20a2* group. (Scale bar = 100 μm). **B** TUNEL assay (number of apoptosis-positive cells/μm) performed on brain hippocampal cross-sections 2 weeks after AAV injection: shRNA-*Slc20a2* (*n* = 5), Scramble (*n* = 5) (Scale bar = 100 μm). **C**–**E** Analysis of neuronal morphology and branching in primary hippocampal neurons transfected with either pSicoR empty vector, shRNA-*Slc20a1*, shRNA-*Slc20a2*, shRNA-*Slc20a2* + WT *SLC20A2*, shRNA-*Slc20a2* + *SLC20A2*-S113A (Pi transport-deficient PiT-2) or shRNA-*Slc20a2* + WT *SLC20A1*. Constructs included an EGFP cassette allowing monitoring of neuronal morphology. **C** Representative images (Scale bar = 20 μm), (**D**) Sholl analysis of 20-59 transfected neurons for each group, from 3 independent neuronal preparations, and (**E**) number and length (μm) of neurites. Results are given as mean ± s.e.m. *p ≤ 0.05, **p ≤ 0.01, ***p ≤ 0.001, ****p < 0.0001, NS: not significant by two-tailed Student’s *t*-test or two-way ANOVA followed by Tukey’s test compared to the control group.

Based on our transcriptional analyses, we further investigated whether *Slc20a2* downregulation could impact HpC function by inducing neuronal death and neuroinflammation. Indeed, we observed that *Slc20a2* downregulation induces a drastic decrease in the number of mature neurons (NeuN^+^) in the CA1, CA2 and CA3 areas. This is associated with a massive increase of the astrocytic marker glial fibrillary acidic protein (GFAP) and the microglial marker ionized calcium-binding adapter molecule 1 (Iba1; encoded by the *Aif1* gene), indicative of increased neuroinflammation (Fig. 3A). TUNEL assays revealed that *Slc20a2* downregulation leads to a significant increase in neuronal apoptosis in the same HpC areas where we observed a decrease in the number of NeuN^+^ neurons (Fig. 3B). In contrast, *Slc20a1* downregulation did not induce any neuronal loss, neither astrocytic/microglial cell increase or neuronal death in the HpC (Fig. 3A). These results demonstrate that hippocampal PiT-2 is essential for neuronal survival and could explain, at least in part, the behavioral phenotypes observed after downregulation of *Slc20a2* in the HpC (Fig. 1E-G and S1G-I).

We next found that a selective *Slc20a2* downregulation in primary HpC neurons decreased dendrite number and length and diminishes dendritic arborization (Fig. 3C-E). These defects were observed from 72 h post transfection, when dendrite number and length was dramatically decreased compared to control-transfected cells (Fig. S3A**)**. Importantly, these phenotypes were rescued after transfection of either wild type (WT) human SLC20A2 or a Pi transport-deficient mutant, SLC20A2-S113A [48], demonstrating that the effect of PiT-2 on neuronal branching is independent of its Pi transport activity (Fig. 3C-E and Fig. S3B-C). It is important to note here that SLC20A2 or SLC20A2-S113A over expression per se had no significant impact on neuronal branching (Fig. S3D). By contrast, we found no morphological changes in primary HpC neurons transfected with shRNA-*Slc20a1* **(**Fig. 3C-E). Moreover, transfection with human WT SLC20A1 was not sufficient to improve the morphological impairments observed after *Slc20a2* downregulation, further illustrating the different and non-redundant roles of PiT-1 and PiT-2 in HpC neurons (Fig. 3C-E). It is known that neuronal morphology, including the degree of dendritic branching, elongation, arborization and coverage of their target area, is crucial for the establishment of appropriate neuronal connectivity, as well as for the sampling and computation of stimuli [49]. Changes in neural circuitry resulting from alterations in dendritic shape and stability can lead to cell death and neuroinflammation [50–52]. Therefore, the decreased neuronal branching phenotype observed after *Slc20a2* downregulation could contribute, at least in part, the neuronal death, neuroinflammation and severe cognitive deficits. Of a note, we also found that *Slc20a2* is not only expressed in neurons but also in astrocyte and microglial cells in the hippocampus (Fig. S3E), which may also suggest a direct role of PiT-2 in these specific cell populations involved in the control of neuroinflammation.

### PiT-1 promotes structural and functional synaptic plasticity in the hippocampus

**Fig. 4:**
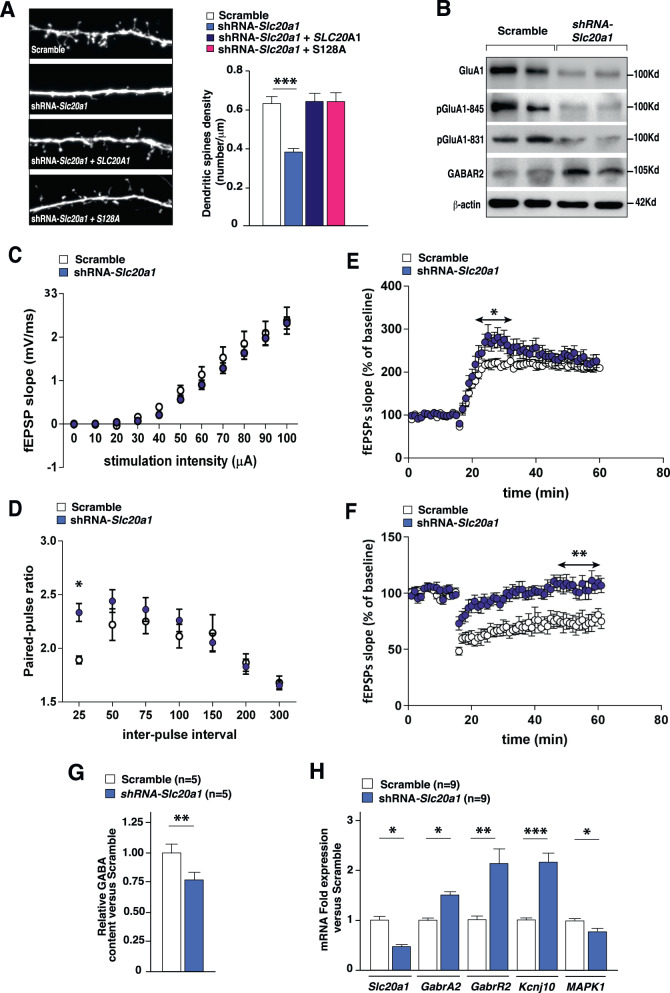
PiT-1 boosts synaptic plasticity and modulates the GABAergic system in the hippocampus. **A** Representative images and quantification of dendritic spine density (number of spines per μm) in primary HpC neurons transfected with either pSicoR empty vector, shRNA-*Slc20a1*, shRNA-*Slc20a1* + WT *SLC20A1* or shRNA-*Slc20a1* + *SLC20A1*-S128A (Pi transport-deficient PiT-1). Analysis was performed on *n* = 25-33 neurons from 3 independent neuronal cultures. EGFP allowed dendritic spine visualization. **B** Western Blot analyses of the total and phosphorylated form of GluA1 (Ser845 and 831) and GABAR2 in HpC injected with either shRNA-*Slc20a1* or Scramble. β-actin was used as a loading control for each sample. Analysis was performed on the HpC of *n* = 5 mice. **C**–**F** Electrophysiological analyses of glutamatergic synaptic transmission in the HpC (CA1 region) performed after selective downregulation of *Slc20a1*. **C** Basal synaptic transmission. The input-output curves were generated by computing field EPSP slope against current intensity in brain slices derived from mice injected with AAV-*Slc20a1*-shRNA (*n* = 8 slices from 4 mice) and mice injected with AAV-Scramble (*n* = 6 slices from 3 mice). I/O curves revealed no differences between the 2 groups. **D** Paired pulse facilitation (PPF) of synaptic transmission. Data were obtained from mice injected with AAV-*Slc20a1*-shRNA (*n* = 10 slices from 4 mice) and group injected with AAV-Scramble-shRNA (*n* = 7 slices from 3 mice). PPF was significantly increased for 25 ms inter-pulse interval with *Slc20a1* downregulation. **E** Long-term potentiation of synaptic transmission. Results show a significant increase of LTP from 20 to 40 min after the beginning of recordings in shRNA-*Slc20a1* slices (*n* = 7 slices from 4 mice) compared to scramble-shRNA slices (*n* = 7 slices from 3 mice). **F** Long-term depression of synaptic transmission. Results show a significant alteration of LTD during the last 10 min of recordings in hippocampal slices after AAV-shRNA-*Slc20a1* injection (*n* = 8 slices from 4 mice) compared to AAV-Scramble-shRNA injected group (*n* = 7 slices from 4 mice). **G** Relative GABA measurements (metabolomics) performed in hippocampi injected with AAV-shRNA-*Slc20a1* by comparison to controls (AAV-Scramble-shRNA). **H** *Slc20a1*, *GabrA2*, *GabrR2*, *Kcnj10*, and *Mapk1* relative expression in hippocampi after local stereotactic injections of either shRNA-*Slc20a1* or Scramble. Results are given as mean ± s.e.m. *p ≤ 0.05, **p ≤ 0.01, ***p ≤ 0.001, ****p < 0.0001, NS: not significant by two-tailed Student’s *t*-test or by non-parametric Mann-Whitney test compared to control group or by two-way ANOVA followed by Sidak’s test compared to control group.

Our behavioral results together with our genomic data strongly suggest that PiT-1 could play a role in maintaining HpC neuronal plasticity by influencing important players of synaptic transmission and plasticity, including proteins involved in synaptic vesicle fusion and membrane trafficking. As shown in Fig. 4A, we found that *Slc20a1* downregulation induced a drastic reduction of dendritic spine density in primary HpC neurons. This reduction was restored after transfection of either human WT SLC20A1 or the Pi transport-deficient mutant SLC20A1-S128A [29] (Fig. 4A and S3B-C). It is important to note here that PiT-1 over expression per se had an impact on synaptogenesis (Fig. S4A). Furthermore, we observed that *Slc20a1* downregulation in the HpC led to a reduction of GluA1 and pGluA1 (at both Ser831 and Ser845), which may induce a modification of synaptic strength (Fig. 4B and S4B). Our electrophysiological analyses on brain slices from AAV-shRNA-injected mice revealed that *Slc20a1* downregulation did not significantly affect basal glutamatergic neurotransmission, defined by input-output (I/O) curves (Fig. 4C), but induced a sustained increase of neuronal facilitation at short inter-pulse intervals, highlighted by paired-pulse facilitation (PPF) ratio analysis, a form of activity-dependent pre-synaptic plasticity (Fig. 4D). These changes in PPF suggest the involvement of PiT-1 in short-term plasticity in HpC neurons. Following stimulation of the Schaffer collateral pathway (between CA1 and CA3 regions) with a Theta-burst protocol to induce long-term potentiation (LTP), we found a rapid and transient rise in the amplitude of the field excitatory postsynaptic potential (fEPSP) in the first phase after LTP induction (Fig. 4E). Changes in LTP induction after *Slc20a1* downregulation in the HpC can reflect a long-lasting change in output in response to a transient input [53]. Moreover, we found a significant blockade of long-term depression (LTD) induced by low-frequency stimulation (Fig. 4F). These results suggest that PiT-1 is required to promote both structural and functional synaptic plasticity and contributes to the regulation of excitability of HpC neurons.

### PiT-1 influences the GABAergic system in hippocampal neurons

Deficits in synaptic plasticity could lead to impairments in neuronal excitability and subsequent CNS pathological states, as short and long-term changes in synaptic efficacy are thought to be important in learning and memory. While excitatory synapses cause the generation, propagation, and potentiation of neuronal responses needed for processing information, inhibitory synapses play an essential role in feedback and feedforward inhibition which control neural excitability [54–58]. Remarkably, unbiased functional enrichment analysis of the most altered transcripts in the HpC showed that *Slc20a1* downregulation alters genes related to the GABAergic system and synaptic vesicle fusion/trafficking (Fig. 2 and S4C). GABA neurotransmitter is known to regulate tonic inhibition of local neurons by reducing excitability. Inhibitory synaptic transmission is mediated primarily by the release of GABA and serves to coordinate the pattern of excitation and the synchronization of neuronal network excitability. Moreover, the increase of the excitatory postsynaptic current (EPSC) in the early phase after LTP induction, as well as the LTD decrease observed in the HpC after *Slc20a1* downregulation could be explained by an alteration of the inhibitory GABAergic system. GABA content in the HpC was reduced around 20% after *Slc20a1* downregulation (Fig. 4G). However, we did not find any modifications in the expression of either *Gad65* or *Gad67*, genes encoding the rate limiting enzymes for GABA production (Fig. S5A). A reduction in GABA content may induce overexpression of GABA receptors at a postsynaptic level as a potential compensatory mechanism. Accordingly, we found an increase in the levels of the GABA_B_ receptor, which is known to activate downstream signal cascades that include decreased probability of transmitter release and increase in pre- and post-synaptic K^+^ conductance [59–61] (Fig. 4H).

### PiT-1 regulates the expression of genes involved in synaptic vesicle fusion and membrane trafficking

**Fig. 5:**
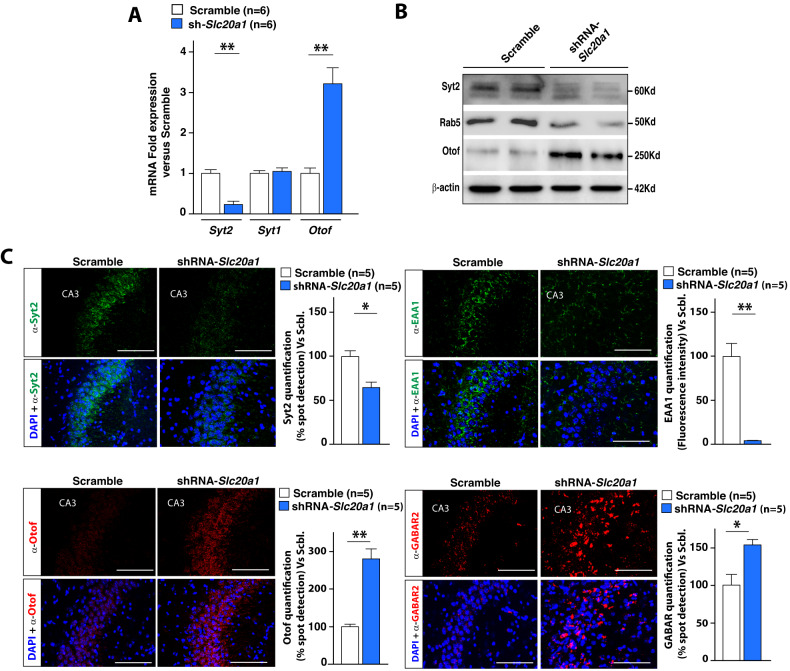
PiT-1 promotes synaptic vesicle fusion and membrane trafficking in hippocampal neurons. **A** Synaptotagmin 2 *(Syt2)*, Synaptotagmin 1 *(Syt1)* and Otoferlin (*Otof*) relative gene expression in the HpC after local stereotactic injections of either Scramble or shRNA-*Slc20a1*. **B** Western Blot analyses of Syt2, Rab5 and Otof in HpC after injection of AAVs expressing either Scramble or shRNA-*Slc20a1*. β-actin was used as a loading control for each sample. **C** Otorferlin, GABAR2, Syt2 and EAA1 immunofluorescence (scale bar = 50 μm) on the HpC CA3 region (*n* = 5) after *Slc20a1* silencing and compared to controls. Brains were collected from two independent cohorts of mice for each group. Results are given as mean ± s.e.m. *p ≤ 0.05, **p ≤ 0.01, ***p ≤ 0.001, ****p < 0.0001, NS: not significant by two-tailed Student’s *t*-test compared to control group.

Presynaptic GABA content can be differentially regulated by changes in synaptic vesicular fusion and transport. This notion together with our RNAseq findings led us to hypothesize that PiT-1 may trigger important functions in synaptic vesicle fusion and membrane trafficking in HpC neurons. When testing this hypothesis, we found that Synaptotagmin 2 (Syt2), a major Ca^2+^ sensor that controls synaptic transmission and synaptic vesicle fusion and mediates fast synchronous GABA release [62, 63], is drastically reduced in CA3 neurons after *Slc20a1* downregulation (Fig. 5A–C and S5B). By contrast, we did not observe any changes in Synaptotagmin 1 (*Syt1*) mRNA expression (Fig. 5A). Along the same line, we also observed a decrease in Rab5 and EAA1, two key players of ER vesicular transport, after *Slc20a1* downregulation (Fig. 5B, C and S5B). These defects in Syt2, Rab5 and EAA1 were accompanied by a significant accumulation of GABA_B_ receptor in both CA3 and CA1 (Fig. 5C and S5C).

### PiT-1 modulates the expression of Otoferlin, a protein implicated in synaptic vesicle trafficking

**Fig. 6:**
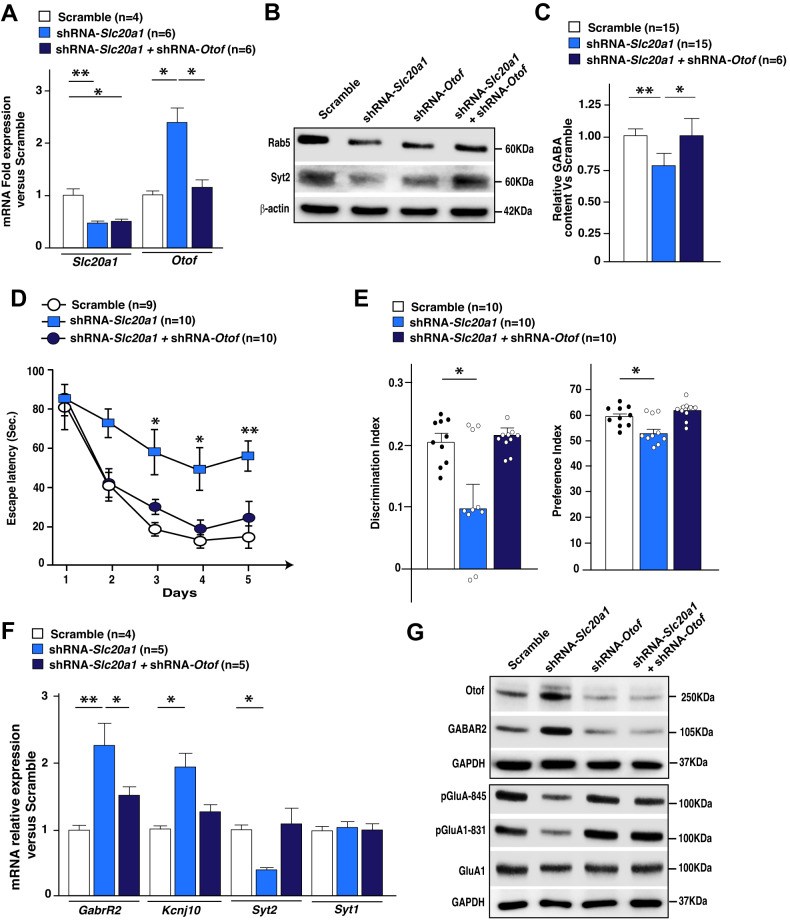
Restoration of Otoferlin levels attenuates the cognitive deficits and membrane trafficking protein perturbations observed after *Slc20a1* downregulation. **A** Relative expression of *Slc20a1* and *Otof* in hippocampi injected with either shRNA-Scramble, shRNA-*Slc20a1*, or shRNA- *Slc20a1*+ shRNA-*Otof*. **B** Western blot analyses of Otof, Syt2 and Rab5 in hippocampi from Scramble (*n* = 4), shRNA-*Slc20a1* (*n* = 4), shRNA-*Otof* (*n* = 5) or shRNA-*Slc20a1* + shRNA-*Otof* (*n* = 5) mice. β-actin was used as a loading control for each sample. **C** Relative GABA measurements performed in Scramble, shRNA-*Slc20a1* or shRNA-*Slc20a1* + shRNA-*Otof*. **D**–**E** Morris Water Maze (**D**) and Novel Object Recognition (NOR) (**E**) was performed in 3-month-old mice injected with either Scramble, shRNA-*Slc20a1* or shRNA-*Slc20a1* + shRNA-*Otof*. **F** *GabrR2*, *Kcnj10*, *Syt2* and *Syt1* relative expression in hippocampi from Scramble, shRNA-*Slc20a1* or shRNA-*Slc20a1* + shRNA-*Otof* - injected mice. All behavioral tests were performed using at least 2 independent animal cohorts. **G** Western blot analyses of Otof, GABAR2, and the total and phosphorylated form of GluA1 (Ser845 and 831) in HpC injected with either Scramble, shRNA-*Slc20a1*, shRNA-*Otof* or shRNA-*Slc20a1* + shRNA-*Otof*. GAPDH was used as a loading control for each sample. Results are given as mean ± s.e.m. *p ≤ 0.05, **p ≤ 0.01, ***p ≤ 0.001 by two-tailed Student’s *t*-test or 2-way ANOVA followed by Tukey’s multiple comparisons test compared to the control group.

Otoferlin (*Otof*), a large multi–C2 domain protein involved in synaptic vesicle fusion and membrane trafficking, was found to be one of the major upregulated genes after *Slc20a1* silencing (Figs. 2E, J, 5A and S5B). Interestingly, it was previously reported that *Otof* overexpression could lead to asynchronous vesicle release and replenishment in neurons [64, 65]. We performed dual stereotactic injections of AAVs expressing shRNA-*Slc20a1* and shRNA-*Otof* and compared them to the ones injected with either scramble or shRNA-*Slc20a1* alone. We first confirmed that shRNA-*Otof* was sufficient to counteract the upregulation of *Otof* in the HpC after *Slc20a1* silencing (Fig. 6A), and that strikingly, preventing the induction of *Otof* improved the deficits in Syt2 and Rab5 protein accumulation, as well as GABA content, observed after *Slc20a1* downregulation in the HpC (Fig. 6B-C and S6A). Furthermore, mice targeted for both *Slc20a1* and *Otof* did not exhibit any memory impairments, as shown in the NOR and MWM behavioral tasks (Fig. 6D-E and S6B-C), contrary to the *Slc20a1*-downregulated mice. This improvement in memory performance was also associated with a restoration of GluA1 phosphorylation and GABA_B_ receptor accumulation levels in the HpC (Fig. 6F-G and S6D). These data demonstrate that PiT-1 influences membrane trafficking and GABAergic system function by modulating Otof levels, and that this regulation is important for the formation of novel memory in the HpC.

## Discussion

In this study, we provide the demonstration that PiT-1 and PiT-2 play key, albeit distinct, roles for the control of hippocampal-dependent memory and neuronal plasticity. We show that *Slc20a1* and *Slc20a2* expression is spatially segregated and associated with independent gene clusters in the HpC, thereby supporting the intriguing possibility that they exert distinct functions. Accordingly, we show that PiT-2 promotes the maintenance of neuronal branching and survival, whereas PiT-1 favors neuronal plasticity in HpC neurons. We also revealed that PiT-1 downregulation leads to impairments in synaptic vesicle trafficking which are associated with an upregulation of Otoferlin, a multi-C2 domain protein involved in the regulation of both exocytosis and clathrin-mediated endocytosis, and deficits in the GABAergic system. These results shed light on the physiological importance of the Na-Pi co-transporters in the brain, pertaining to the control of neuronal functions and cognition.

Despite a general consensus that *Slc20a* genes are ubiquitously expressed throughout the body, findings so far suggest that they are not equally omnipresent across the different organs, and that their levels vary significantly within different tissues and specific cell types [41, 66]. Our study indicates that the levels of expression of both *Slc20a* genes are significantly higher in the brain compared to other organs. Moreover, we found that *Slc20a1* and *Slc20a2* are not expressed in the same brain areas. We found that *Slc20a1* is highly expressed in the CA3, while *Slc20a2* is predominantly expressed in the CA1 and DG. This differential localization is also associated with distinct HpC-dependent functions. We demonstrate that although both *Slc20a1* and *Slc20a2* impact spatial and episodic memory, only *Slc20a1* affects contextual memory, while only *Slc20a2* is linked to anxiety-related phenotypes.

The ability of PiT-1 and PiT-2 to transport Pi across the plasma membrane was the first described function for these membrane proteins [26, 27]. Since then, multiple other cellular and physiological functions have been reported, independent to its Pi transport function [29, 30, 33–39, 67]. Most of the reported Pi transport-independent functions of PiT-1 provide insight into the functional multiplicity of this protein, establishing it as a molecular hub modulating the availability or function of specific proteins. For instance, we have previously shown that PiT-1 modulates the degradation of IRS1 in hepatocytes by binding to the deubiquitinase USP7, thereby modulating glucose metabolism and insulin signaling [36]. Similarly, the interaction of PiT-1 with protein disulfide isomerase (PDI) in growth-plate chondrocytes is essential for proper ER function [35]. The present study shows that the cellular effects originating from *Slc20a1* downregulation occur without altering Pi transport in HpC neurons. Interestingly, this cannot be explained by a possible functional compensation by PiT-2, as its expression levels remain unchanged. In contrast, downregulation of *Slc20a2* in HpC neurons does affect Pi transport. These observations suggest that, unlike PiT-2, PiT-1 could be present in intracellular membranes of vesicular compartments, as it has been shown in other cell types [35, 45]. Moreover, PiT-2 effects may not only be linked to its Pi transport activity, as suggested by the rescue of defects in neuronal morphology by a PiT-2 Pi-transport-deficient mutant. Overall, our data show that both PiT-1 and PiT-2 have Pi transport-independent functions in the brain which are crucial for the maintenance of neuronal integrity and plasticity.

Heterozygous mutations of *SLC20A2* have been identified in primary familial brain calcification (PFBC), an inherited form of neurological disorders characterized by cognitive impairments [14–18]. To date, pathogenic variants in six genes have been related to PFBC, among which *SLC20A2* is the most frequently affected gene (62% of identified mutations) [14–18]. All PFBC patients display symmetrical and bilateral calcification of the basal ganglia, but a recent systematic review of the literature indicates that only 60% of the patients carrying *SLC20A2* mutations are symptomatic, manifesting both motor and non-motor clinical symptoms, including cognitive deficits, such as memory defects [14–18]. However, the etiology of brain calcification in PFBC patients is poorly described and cannot be simply explained by a defect in Pi transport in the brain. Indeed, PFBC patients carrying *SLC20A2* mutations have higher Pi levels in their cerebrospinal fluid, but this is not necessarily the case for other PFBC patients, despite the presence of brain calcification [66]. This observation challenges the established link between Pi transport and mineralization mechanisms, as we have recently illustrated in the bone [68]. Interestingly, we could detect a slight increase of extracellular Pi concentration in the culture medium of neurons downregulated for *Slc20a2*, which is consistent with the decreased Pi transport in *shRNA Slc20a2* neurons (Fig. 1D) and the role of PiT-2 as a Pi transporter. However, the observed increase is very low and we could not detect any calcium-phosphate crystal formation in these conditions. This result is consistent with in vivo data showing that brain calcification would only occur when Pi CSF largely increases (up to 2 mM). The absence of crystal formation in our neuron culture is also consistent with thorough in vitro studies demonstrating the absence of calcification at 1 mM Pi and the need to increase Pi concentration to at least 1.8 mM to initiate these events [42–44]. Moreover, since both PiT-1 and PiT-2 are required for Pi sensing function [67], a greater similarity in the observed phenotypes would have been expected in this study if Pi sensing was involved in the observed phenotypes. Consistent with this, we have shown here that the phenotypic consequences of PiT-1 or PiT-2 downregulation are different. Therefore, our experiments collectively argue in favor of a global PiT-2 effect independent of Pi in neurons, without it being possible to distinguish at this stage what is transport-related and what is sensing-related. These results also illustrate that the mechanisms leading to a PFBC-like phenotype following PiT-2 deletion are likely to be more complex than originally thought.

We found that *Slc20a2* downregulation induces significant neuroinflammation in the HpC, mainly reflecting a neurotoxic astrocyte response. Mounting data underscore the importance of neuroinflammation as a key element in the pathophysiology of PFBC, as well as several neurodegenerative and neuropsychiatric disorders, such as anxiety and depression [17, 69–71]. Activation of glial cells, with the ensuing neuroinflammatory cascades that include the release of various soluble factors, can further accelerate the rate of neurodegeneration, synaptic loss and synaptic dysfunction associated with neurological disorders [72, 73]. This increase in neuroinflammation could be explained both by a direct, as well as, an indirect *Slc20a2* downregulation effect, stemming from deficits in neuronal integrity and survival. Indeed, we observed that *Slc20a2* downregulation in neuron induces a reduction of neuronal branching and survival. This is also consistent with a study in *Drosophila*, where disruption of PiT-2 interaction with the cytoskeleton protein MAP1B induced a similar branching phenotype [74]. Neuronal cell death is known to be associated with neuroinflammation and as a pathological hallmark of cognitive disorders and neurodegenerative diseases [75–77]. Moreover, the induction of neuroinflammation and neuronal death in the HpC after *Slc20a2* downregulation could also explain, at least in part, the exploratory- and anxiety-like behavioral phenotypes observed in these mice. Indeed, several reports indicate that indicate that neuroinflammation is a risk factor for severe stress-induced anxiety [78]. Interestingly, we could also detect presence of *Slc20a2* transcripts in both astrocytes and microglial cells in the hippocampus, which may suggest a role of PiT-2 in these two specific cell populations and could provide additional explanations for the increase in hippocampal neuroinflammation observed after *Slc20a2* downregulation. Importantly, drugs with microglia-targeted anti-inflammatory properties represent novel alternative or complementary anxiolytic therapeutic approaches [79, 80], with novel therapeutic perspectives for PiT-2 manipulation.

By contrast, *Slc20a1* downregulation in the HpC does not lead to neither neuronal cell death nor neuroinflammation but induces a selective decrease in neuronal plasticity without affecting basal neurotransmission. This defect is observed at the structural (large reduction in dendritic spine density) and functional (LTP and LTD) levels. *Slc20a1* downregulation blocks low frequency stimulation of Schaffer collaterals induced LTD. Since LTD requires a persistent dephosphorylation of the GluA1 subunit of AMPA receptors at serine 845 [81], the defects in LTD maintenance observed in our mouse model could be constitutive to a reduction of both GluA1 and pGluA1 (at both Ser831 and Ser845) through an occlusive mechanism. Second, *Slc20a1* downregulation also leads to an increase in early stage LTP evoked by theta-burst stimulation. This protocol induces a long-lasting enhancement of inhibition that may dampen increases in the excitability CA1 neurons following tetanization [82]. Thus, molecular perturbations within inhibitory circuits could explain the rise of fEPSP slopes in the first phase after LTP induction. Consistent with this hypothesis, we found a decrease in GABA content in the HpC of *Slc20a1*-downregulated mice without any changes in the expression levels of the genes encoding the GABA rate limiting enzymes *Gad65* and *Gad67*, suggesting a decrease in GABA release rather than biosynthesis. In addition, we found a significant accumulation of GABA_B_ receptor in the HpC after *Slc20a1* downregulation, pointing to a likely alteration in the turnover mechanism of GABA_B_ receptor. Indeed, GABA_B_ receptor accumulation could be a compensatory mechanism induced by the decrease in GABA levels, but also by defects in GABA_B_ internalization. These perturbations in the inhibitory network associated with the strong reduction of dendritic spine density and GluA1 subunit levels could explain the lack of differences in the I/O curve.

The accumulation of GABA_B_ receptors critically depends on the residence time at the cell surface and finally the rates of endocytosis and degradation [83–85]. Endocytosis of GABA_B_ receptors involves their recruitment into clathrin-coated pits and their internalization in a dynamin-dependent manner [86–88]. In our experimental conditions, *Slc20a1* downregulation led to lower levels of key players involved in Ca2^+^-dependent membrane trafficking and ER vesicular transport, such as Rab5, Syt-2 and EAA1. Taken together, these observations suggest a role of PiT-1 in the control of vesicular trafficking in HpC neurons, which could be linked to the regulation of GABAergic inhibitory system.

Neuroinflammation and alteration of GABAergic system function are known to be frequently associated with neurodegenerative diseases (such as Alzheimer’s, Parkinsons’, ALS, etc.) [69–71, 89]. Moreover, among the modulated genes observed after PiT-1 and PiT-2 downregulation, several are of interest in the context of neurodegenerative diseases. Indeed, CX3CR1 is constitutively expressed in human CNS astrocytes and microglial cells in multiple sclerosis (MS) patients [90]. Increasing evidence also suggests that colony stimulating factor 1 receptor (CSF1R) is required for the development, maintenance and proliferation of microglia, at least in rodents [91]. Collectively, alterations in survival gene expression by *Slc20a2* downregulation, and neuronal plasticity and GABAergic system genes by *Slc20a1* downregulation, suggest that these proteins impact different aspects of cell homeostasis in the HpC.

We found that one of the most upregulated genes in the HpC after PiT-1 downregulation is *Otoferlin* (*Otof*), a multi-C2 domain protein involved in multiple functions in regulating vesicle cycling, including both exocytosis and clathrin-mediated endocytosis. Otof was originally described as abundantly expressed in sensory inner hair cells (IHCs) of the cochlea, acting as a key player for the regulation of the final steps of synaptic vesicle fusion at IHC ribbon synapses [92]. *OTOF* mutations lead to autosomal recessive non-syndromic prelingual deafness DFNB9 [93] or hearing impairment that can be temperature sensitive [94]. However, the role of Otof in the HpC remains largely unknown. Here, we found that upregulation of Otof after *Slc20a1* downregulation is associated with a decrease in the levels of Ca^2+^-dependent membrane trafficking proteins, such as Rab5, Syt-2 and EAA1 and a perturbation of the GABAergic system. Importantly, this link is further reinforced by the fact that restoration of Otof levels in *Slc20a1*-downregulated HpC is sufficient to improve Rab5, Syt-2 and EAA1 levels, GABA content and GABA_B_ receptor accumulation, as well as memory deficits. This set of results suggest that, by repressing Otof levels, PiT-1 controls synaptic vesicle trafficking, GABAergic system maintenance and, ultimately, cognitive fitness.

In summary, our results open a new field of exploration for PiT-1 and PiT-2 in the CNS. A better understanding of the exact mode of action of PiT-1 and PiT-2 in regulating membrane trafficking and neuronal death in HpC neurons will provide the foundation for the development of novel effective therapies for neurodegenerative diseases and neurological disorders.

## Material and methods

### Animal housing and ethics

All experiments were performed on C57BL/6 J wild type (WT) male mice obtained from Janvier Laboratory. All mice were 3 months of age at the onset of the experiments. Habituation of the mice in our housing facility before any behavioral assessment was 2 weeks. Mice were housed 5 per cage, in polycarbonate cages (35.5 × 18 × 12.5 cm), under a 12-h light/dark cycle with *ad libitum* access to food and water. All behavioral experiments were performed between 10AM and 5PM. In all experiments, animals were randomly assigned to experimental groups. Group sizes were determined after performing a power calculation to lead to an 80% chance of detecting a significant difference (*P* ≤ 0.05). All behavioral experiments were performed in accordance with the European Communities for Experimental animal use (2010/63/EU) and local ethical committee review procedures and protocols (APAFIS # 19144).

### Stereotaxic surgery

Mice were anesthetized by intraperitoneal injection of ketamine hydrochloride (20 mg/ml BW) (1000 Virbac) and xylazine (100 mg/ml BW) (Rompun 2%; Bayer) and placed in a stereotaxic frame (900SL-KOPF). Ophthalmic eye ointment was applied to the cornea to prevent desiccation during surgery. The area around the incision was trimmed and an iodine solution was applied (Vétédine). AAVs were injected bilaterally into the dorsal hippocampus using the following coordinates (from Bregma, Paxinos and Franklin, 2008): X = +/−1.4 mm, Y = 2.0 mm and Z = −1.33 mm. A 1 μl volume of either AAV was injected bilaterally (0.25 μl/min). To limit reflux along the injection track, the needle was maintained in situ for 4 min between each individual injection.

### Adeno-associated viruses expressing shRNA

Adeno-associated viruses (AAV) expressing shRNA were purchased from Vector Biosystems Inc. (Malvern PA). shRNAs specific to mouse PiT-1 (*Slc20a1*) (AAV9-GFP-U6-*mSlc20a1*-shRNA), mouse PiT-2 (*Slc20a2*) (AAV9-GFP-U6-*mSlc20a2*-shRNA), mouse Otoferlin (*Otof*) (AAV9-GFP-U6-m*Otof*-shRNA) or scrambled non-targeting negative control (AAV-GFP-U6-scrmb-shRNA) were injected 3 weeks prior to behavioral tests or brain tissue collection. The AAV titers were between 2 and 5 × 10^13^ GC/ml.

### Behavioral assessment

For all behavioral assessments, mice were maintained in the testing room for at least 2 h prior to testing. Behavior was scored by two observers blind to the groups.

#### Novel object recognition paradigm (NOR)

The testing arena consisted of a plastic box (60 × 40 × 32 cm). Mice could not see each other during the experiment. Two different objects were used: (A) a blue ceramic pot (diameter 6.5 cm, maximal height 7.5 cm) and (B) a clear/plastic funnel (diameter 8.5 cm, maximal height 8.5 cm). The objects elicited equal levels of exploration as determined in control experiments and training phases. Mice were placed in the center of the arena at the start of each exposure. Sessions were recorded with a video camera. The NOR paradigm consisted of three phases over 3 days. On day 1 (habituation phase), mice were given 5 min to explore the arena devoid of objects and were then taken back to their home cage. On day 2 (training phase), mice were allowed to explore for 10 min two identical objects arranged in a symmetric opposite position from the center of the arena and were then transported to their home cage. On day 3 (testing phase), mice were given 15 min to explore two objects: a familiar object and a novel one, in the same arena, keeping the same object location. Between exposures, arenas were cleaned with a disinfectant (Phagospore), and the bedding was replaced. The following behaviors were considered as exploration of the objects: sniffing or touching the object with the nose or with the front legs or directing the nose to the object at ≤ 1 cm distance. Exploration was not scored if the mouse was on top of the object or completely immobile. Total exploration time was quantified during the training and testing phases. The preference index (time spent exploring the new object / the total time spent exploring both objects) and the discrimination index (time spent exploring the new object - time spent exploring the familiar object) / (total time spent exploring both objects) were calculated. As a control, the preference index for the (right/left) object location or for the object A versus B during the training phase of the NOR was measured.

#### Contextual fear conditioning (CFC)

Conditioning chambers (Bioseb, France) were located inside larger, insulated plastic cabinets (67 × 55 × 50 cm), and mice were tested individually. The floor of the chambers consisted of 27 stainless steel bars wired to a shock generator with scrambler for the delivery of foot shock. The signal generated by movement was recorded and analyzed through a high sensitivity weight transducer system. The analog signal was transmitted to the Freezing software module through the load cell unit for recording purposes and analysis of time active / time immobile (Freezing) was performed. The CFC procedure took place over two consecutive days. On day 1 (training), mice were placed in the conditioning chamber and received 3 foot-shocks (1 s, 0.5 mA), which were administrated at 60, 120 and 180 s post-entry into the chamber. On day 2 (testing), contextual fear memory was assessed 24 h after training, by returning the mice to the conditioning chamber and measuring freezing behavior during a 4 min retention test. Freezing behavior (for a period of at least 2 s) was scored and analyzed automatically using Packwin 2.0 software (Bioseb, France).

#### Morris Water Maze (MWM)

The apparatus was a white circular water tank (diameter: 200 cm, walls: 60 cm high), located in a room with various distal cues. The tank was filled with water (depth: 50 cm) maintained at 22 °C ± 1 °C, which was made opaque by the addition of a nontoxic white paint. A 12 cm round platform was hidden 1 cm below the water surface. Extra maze geometric and high-contrast cues were mounted on the walls of the tank with the ceiling providing illumination. Data was collected using a video camera fixed to the ceiling and connected to a video tracking system (Anymaze). Each daily trial consisted of four swimming sessions, in which each mouse was placed in the tank facing the wall and was allowed to swim for 120 s. A trial terminated when the animal reached the platform, where it remained for 5 s. Mice were removed and placed back in their home cages for a 5 min inter-trial interval. To prevent hypothermia, the animals were gently dried with a paper towel between and after the trials. Different release points were used from day to day. Swimming time to the platform was calculated as the latency to reach the platform. Animal movements were recorded using Anymaze to calculate parameters of the performance of mice. Mice floating in all trials were removed from the analysis.

#### Light-to-Dark Transition test (D/LT)

This test is based on the innate aversion of rodents to brightly illuminated areas and on their spontaneous exploratory behavior in response to the stressor that light represents. The test apparatus consists of a dark, safe compartment and a brightly illuminated (1000 lx), aversive one. Mice were placed individually in the testing chamber in the middle of the dark area facing away from the doorway to the light compartment. Mice were tested for 10 min, and two parameters were recorded: time spent in the lit compartment and the number of transitions between compartments, indicative of anxiety-related behavior and exploratory activity. Behavior was scored using an infrared light beam activity monitor using actiMot2 Software (PhenoMaster Software, TSE).

#### Open Field Test (OFT)

This test is based on the aversion of rodents to brightly lit areas. Each mouse was placed in the center of the OFT chamber (43 × 43 cm) and allowed to explore for 30 min. Mice were monitored throughout each test session by infrared light beam activity monitor using actiMot2 Software (PhenoMaster Software, TSE). Overall ambulatory activity was quantified as the total distance traveled. Anxiety-like behavior was assessed by measuring the time and distance spent in the center (aversive) versus the periphery of the open-field chamber.

### Immunofluorescence on brain sections

Mice were deeply anesthetized with a mixture ketamine/xylazine and transcardially perfused with cold PBS, followed by cold 4%PFA. Brains were post-fixed overnight in 4% PFA at 4 °C. 30 μm serial coronal floating sections were obtained using a vibratome. For single immunofluorescence, sections were blocked with 10% donkey serum for 30 min at room temperature and then incubated with either mouse anti-NeuN (1:500, Millipore), mouse anti-GFAP (1:400, Abcam), rabbit anti-IbaI (1:500, WAKO), rabbit anti-Synaptotagmin 2 (Syt2) (1:400, Novusbio), rabbit anti-Otoferlin (Otof) (1:400, Arigo Laboratories), mouse anti-EAA1 (1:200, BD Transduction laboratories), rabbit anti-GABAR (1:400, Arigo Laboratories), overnight at 4 °C. The sections were washed with PBS before and after being incubated with Cy3-, Cy5- or Alexa-conjugated secondary antibodies (1/200, Jackson Immunoresearch) for 2 h at room temperature in blocking buffer. All sections were mounted onto gelatin-subbed slides and cover-slipped using Mowiol with DAPI. Images were obtained using a Zeiss Apotome2 fluorescence microscope and analyzed with ImageJ software.

### Von Kossa staining

Primary HpC neurons cultures were prepared, plated and infected (MOI5) at DIV1 with lentiviruses (pSicoR) expressing either shRNA targeting mouse PiT-2 (*Slc20a2*) or shRNA Scramble, as described above. At DIV15 the cultured medium was collected for measurement of extracellular Pi concentration and the neurons were washed 3 times with PBS1X and fixed 1 h with cold ethanol 70%. Then, Von Kossa staining was performed as described in [95].

### Pi measurement in culture medium

The culture medium of primary HpC neurons infected with lentiviruses (pSicoR) expressing shRNA targeting either mouse PiT-2 (*Slc20a2*) was collected at DIV15. Measurement of the extracellular Pi concentration was performed using the Olympus AU400 Automated Clinical Chemistry Analyzer (Beckman Coulter).

### TUNEL assay

Two weeks after AAVs stereotactic injections, 30 μm cryostat brain sections were processed using the ApopTag® Peroxidase Direct In Situ Apoptosis Detection Kit (Millipore) according to the manufacturer’s protocol. Images were obtained using a Nanozoomer 2.0 (Hamamatsu) and the number of apoptotic cells was counted on 14 serial HpC sections for each mouse.

### In situ hybridization

*Slc20a1* and *Slc20a2* mRNA in-situ hybridization on mouse brain sections was performed with RNAscope Multiplex Fluorescent assay and ACD HybEZ™ II Hybridization System (Biotechne). Mice were transcardially perfused with cold PBS, followed by cold 4% PFA as described above. Brains were post-fixed 24 h in 4% PFA, cryoprotected in 20% sucrose for 24 h at 4 °C, embedded in cryo-embedding medium (OCT), and frozen. 10 μm brain sections were processed according to manufacturers’ instructions. RNAscope probes Mm-Slc20a1 (Cat. No. 417171), Mm-Slc20a2-C2 (Cat. No. 417151-C2), Mm-Gfap (Cat. No. 319141) and Mm-Iba1 (Cat. No. 313211) were visualized with Opal™ 520 and Opal™ 540 Reagents (PerkinElmer), respectively.

### Targeted LC/MS GABA analyses

For GABA metabolomic analysis metabolites were extracted using solution composed of 50% methanol, 30% acetonitrile (ACN) and 20% water. The volume of the extraction solution was adjusted to weight of the tissue (1 ml per 50 mg). After addition of extraction solution, samples were vortexed for 5 min at 4 °C and centrifuged at 16,000 g for 15 min at 4 °C. The supernatants were collected and stored at −80 °C until analysis. LC/MS analyses were conducted on a QExactive Plus Orbitrap mass spectrometer equipped with an Ion Max source and a HESI II probe coupled to a Dionex UltiMate 3000 UPLC system (Thermo). The 5 µl samples were injected onto a ZIC-pHILIC column (150 mm × 2.1 mm; i.d. 5 µm) with a guard column (20 mm × 2.1 mm; i.d. 5 µm) (Millipore) for LC separation. Buffer A was 20 mM ammonium carbonate, 0.1% ammonium hydroxide (pH 9.2), and buffer B was ACN. The chromatographic gradient was run at a flow rate of 0.2 µl min−1 as follows: 0–20 min, linear gradient from 80% to 20% of buffer B; 20–20.5 min, linear gradient from 20% to 80% of buffer B; 20.5–28 min, 80% buffer B. The mass spectrometer was operated in full scan, polarity switching mode with the spray voltage set to 2.5 kV and the heated capillary held at 320 °C. The sheath gas flow was set to 20 units, the auxiliary gas flow to 5 units and the sweep gas flow to 0 units. The metabolites were detected across a mass range of 75–1000 m/z at a resolution of 35,000 (at 200 m/z) with the automatic gain control target at 106 and the maximum injection time at 250 ms. Lock masses were used to ensure mass accuracy below 5 ppm. Data were acquired with Thermo Xcalibur software (Thermo). The peak area of GABA was determined using Thermo TraceFinder software (Thermo), identified by the exact mass of each singly charged ion and by the known retention time on the HPLC column.

### Semi-quantitative RT-qPCR

Following dissection, tissues were immediately snap-frozen and maintained in −80°C until use. Total RNA was isolated with TRIzol Reagent using a homogenizer. Single-strand cDNA was synthesized from total RNA (2 μg) by using SuperScript II Reverse Transcriptase. RT–qPCR was performed in triplicate for each sample using iTAQ SYBR Green (BioRad). The following primers were used: *Slc20a1* primers (5’-cttccttgttcgtgcgttcat-3’ and 5’-aagaggttgattccgattgtgc-3’), *Slc20a2* primers (5’-tggacgggtatctgtggatg-3’ and 5’-ccgactgaaaacgccaggat-3’), *Mapk1* primers (5’-ggttgttcccaaatgctgact -3’ and 5’-caacttcaatcctcttgtgaggg-3’), *Syt1* primers (5’-gacaaaagtccaccggaaaacc-3’ and 5’- ccagtgtcttgccacctaattc-3’), *Syt2* primers (5’-gccataccaggagttaggagg-3’ and 5’-ctccccgatgatgtcatgct-3’), *Kcnj10* primers (5’-gtcggtcgctaaggtctattaca-3’ and 5’-ggccgtctttcgtgaggac-3’), *Otof* primers (5’-cagcatggccctgattgttc-3’ and 5’-aagactgccctcggaaagtg-3’), *Grm4* primers (5’-cccatacccattgtcaagttgg-3’ and 5’-tgtagcgcacaaaagtgacca-3’), *GabrA2* primers (5’-ggaagctacgcttacacaacc-3’ and 5’-ccatcgggagcaacctgaa-3’), *GabrR2* primers (5’-ggcatcttgcccattaaaagtcc-3’ and 5’-accaaagcgagtctcatcaaat-3’), *Atf4* primers (5’-atgatggcttggccagtg -3’ and 5’-ccattttctccaacatccaatc-3’), *Slc17a1* primers (5’-agtcgagctaaaagtcttcctg-3’ and 5’-actttcttggggaggcactg-3’), *Slc17a2* primers (5’-tctgctgtgtcctgtggttc-3’ and 5’-tgggagaactcgactgctga-3’), *Slc17a3* primers (5’-agcctgtccagaggaaacac-3’ and 5’-tgtgacgaaggcgattccat-3’), *Slc17a4* primers (5’-ccaaggcatggctcaggtta-3’ and 5’-tgcagcgatggtgatgagtt-3’), *Slc34a1* primers (5’-tgacttcaggcgggctttt-3’ and 5’-gacatggtgtaggtagcccg-3’), *Slc34a2* primers (5’-gtggctggactggtgatagg-3’ and 5’-ccgaaccgtcagcaatgaag-3’), *Slc34a3* primers (5’-cctcaccatacatgcagagcta-3’ and 5’-ggcatccagagtagggttgg-3’), *Gad65* primers (5’-tccggcttttggtccttcg-3’ and 5’-atgccgcccgtgaactttt-3’), *Gad67* primers (5’-ccaccaaggttctggatttcc-3’ and 5’-gtacttcagggtgtctctacagt -3’). *Gapdh* was used as reference (5’-aggtcggtgtgaacggat-3’ and 5’-ggggtcgttgatggcaaca-3’).

### Western blot analysis

Hippocampi were dissected, snap-frozen and homogenized in RIPA lysis buffer (25 mM Tris HCl, pH 7.6, 150 mM NaCl, 1% NP40, 1% Na deoxycholate, 0.1% SDS and cOmplete protease and phosphatase inhibitors (Roche)). The lysates were loaded on a 4-12% SDS polyacrylamide gradient gel and transferred onto a PVDF membrane. The blots were blocked in Tris-buffered saline with Tween (TBST)-5% BSA and incubated with either mouse anti-β-actin (1:5000, A-2228: Sigma), mouse anti-GAPDH (1:5000, MAB374: Merck), rabbit anti-Otof (1:1000, ARG59222: Arigo Laboratories), rabbit anti-Syt2 (1:1000, NB100-91297: NovusBio), mouse anti-Rab5 (1:1000, 21433, Cell Signaling), mouse anti-GluR1 (1:1000, MAB2263: Merck), rabbit anti-pGluR1 (S845) (1:1000, 8084: Cell Signaling), mouse anti-pGluR1 (S8431) (1:1000, 04823: Merck), rabbit anti-GABAR2 (1:500, Arigo Laboratories). Horseradish peroxidase–conjugated secondary antibodies (anti-mouse IgG), HRP-linked antibody (7076, Cell Signaling) and anti-rabbit IgG, HRP-linked antibody (7074: Cell Signaling) and revealed using an ECL kit (Clarity Western ECL Substrate: BioRad) for protein detection. Selected films were scanned and quantified using BioRad Image Lab software (Version 5.2). β-actin or GAPDH were used for normalization.

### Primary cultures of hippocampal neurons

HpC neurons were isolated from mouse embryos (embryonic day 16.5). After dissection, the tissue was digested with trypsin 0.05% and EDTA 0.02% for 15 min at 37 °C. After three washes with DMEM (61965059: Thermo Fisher Scientific) supplemented with 10% FBS, 100 U/ml penicillin-streptomycin and 1x GlutaMAX (Thermo Fisher Scientific), cells were dissociated by pipetting up and down, and then plated. The dissociated cells were plated onto poly-L-lysine-coated plastic plates or glass coverslips for microscopic examination. 24 h after plating, the media was replaced with Neurobasal medium (Thermo Fisher Scientific) containing B27 supplement (Thermo Fisher Scientific), GlutaMAX and Mycozap (Lonza). Half of the media volume was changed two times per week and neurons were maintained in 5% CO2 and 37 °C. Experiments were performed on cells after 18 days in vitro (DIV).

### Lentiviral transduction and Na-dependent Pi uptake in vitro

Primary HpC neurons were transduced (MOI5) at DIV1 with lentiviruses (pSicoR) expressing shRNA targeting either mouse PiT-1 (*Slc20a1*): 5’-tcgagccggcccattgtattgtcggtgcaattcaagagattgca ccgacaatacaatgggtttttgg-3’ (sense); 3’-gatcccaaaaaacccattgtattgtcggtgcaatctcttgaattgcaccgacaatac aatgggccggc-5’(anti-sense); mouse PiT-2 (*Slc20a2*): 5’-tccacagctcatcttccagaatttcaagagaattctggaa gatgagctgtggttttttc-3’ (sense); 3’-tcgagaaaaaaccacagctcatcttccagaattctcttgaaattctggaagatgagctgt gga-5’(anti-sense), or both. At DIV18, cells were washed and incubated in uptake medium containing 100 μm [^32^P]KH_2_PO_4_ (0.5 μCi/ml) in the presence of 137 mm NaCl (total P_i_ transport) or *N*-methyl-d-glucamine (sodium-independent transport) at 37 °C for 10 min. Sodium-dependent P_i_ transport was calculated as the difference between total and sodium-independent P_i_ transports. Cells were washed three times and lysed with 0.1 m NaOH solution. Aliquots of cell lysates were taken for the determination of protein content (Pierce BCA protein assay kit, Thermo Fisher Scientific) and the radioactivity by liquid scintillation counting.

### Transfection of primary hippocampal neurons

Lentiviral plasmid constructs or empty pSicoR plasmid were used to transfect neurons at DIV11 with Lipofectamine 2000 (Thermo Fisher Scientific) following manufacturer’s instructions. For rescue experiments, pSicoR plasmid were co-transfected with the following human SLC20A1 and SLC20A2 constructs (Origene): pCMV6-AC SLC20A1 and pCMV6-Entry SLC20A2. Site-directed mutagenesis was used to obtain the transport-deficient mutants SLC20A1-S128A and SLC20A2-S113A. Neurons were fixed at DIV18 (unless otherwise stated) in 4% PFA / 4% glucose in PBS 1X for 20 min at room temperature. Immunofluorescence using rabbit-anti PiT-1 and PiT-2 (Proteintech) was performed to visualize co-transfected neurons when appropriate. The coverslips were then washed 3 times in PBS and mounted with Fluoromount™ Aqueous Mounting Medium to study dendrite morphology and dendritic spines density.

### Dendrite branching and dendritic spine density analysis in vitro

Images of EGFP-positive neurons were obtained using a Zeiss Apotome2 (20X objective). Dendrites on 8-bit images were traced using the NeuronJ plugin for ImageJ (NIH, Bethesda, MD). For each neuron, tracings were counted, measured, and analyzed using Scholl analysis function [96]. Data was exported to Excel, and GraphPad Prism was used for all statistical analyses. For analysis of Sholl curves, two-way ANOVA was used followed by Bonferroni Multiple Comparisons test. For analysis of dendrite counts and length, Student *t*-tests were used. All tracings and analyses were performed with the experimenter blind to the experimental group.

To determine the dendritic spine density, images of EGFP-positive neurons were obtained using a Zeiss Apotome2 (40X objective). Dendritic spine density was analyzed using NeuronStudio software [97]. For each neuron, spines from two distinct secondary and tertiary dendrite segments were counted. Blind analyses were performed by two independent investigators.

### Electrophysiology

All recordings were carried out by researchers blind to the experimental conditions, (3-month-old mice) stereotactically injected (3 weeks before sacrifice) with either Scramble or shRNA-*Slc20a1* were perfused with cold artificial cerebrospinal fluid (aCSF) containing (in mM): 128 NaCl, 3 KCl, 1.25 NaH2PO4, 10 D-glucose, 24 NaHCO3, 2 CaCl2, and 2 MgCl2 (oxygenated with 95% O2 and 5% CO2, pH 7.35, 295–305 mOsm). Acute brain slices containing the CA3 were cut using a microslicer (DTK-1000, Ted Pella) in sucrose-ACSF, which was derived by fully replacing NaCl with 254 mM sucrose, and saturated by 95% O2 and 5% CO2. Slices were maintained in the holding chamber for 1 h at 37 °C. Slices were transferred into a recording chamber fitted with a constant flow rate of aCSF equilibrated with 95% O2/5% CO (2.5 ml/min) and at 35 °C. Glass microelectrodes (2–4 MΩ) filled with an internal solution containing (mM): 115 potassium gluconate, 20 KCl, 1.5 MgCl2, 10 phosphocreatine, 10 HEPES, 2 magnesium ATP, and 0.5 GTP (pH 7.2, 285 mOsm). All recordings were performed in GFP labeled cells. Cell excitability was measured with 2 s incremental steps of current injections (50, 100, 150, and 200 pA) at −70 mV holding potential. Series resistance was monitored during all recordings at the beginning and end of each recording and data were rejected if values changed by >20%. All data acquisition and on-line analysis of firing rate were collected using 700B amplifier, Digidata 1322 A digitizer and pClamp 10.2 (Molecular Devices). Spontaneous excitatory postsynaptic currents (sEPSCs) were recorded in voltage clamp at a holding potential of −70 mV with series resistance of <6 MΩ, in the presence of picrotoxin (50 μM). sEPSC was analyzed with the MiniAnalysis software (Synaptosoft). Briefly, sEPSCs were detected automatically using an amplitude threshold of 10 pA and then visually accepted or rejected based upon the rise and decay times. For recording sEPSCs, the external aCSF solutions contained 50 μM dl-2-amino-5-phosphonovaleric acid (AP-5) to block NMDA receptors. The ionic composition of the internal (pipette) solution for voltage clamp studies of sEPSC consisted of (in mM) 140 CsCl, 10 phosphocreatine, 2 MgCl2, 10 EGTA, 2 magnesium ATP, and 0.5 GTP and 10 HEPES with a pH adjusted with CSOH.

#### Ex vivo slice preparation

Brain slices were prepared from 3-month-old mice stereotactically injected (3 weeks before sacrifice) with either Scramble or shRNA-*Slc20a1*. The brain was removed quickly and 350 μm thick sagittal slices containing both cortex and hippocampus were cut in ice-cold sucrose solution (in mM: KCl 2.5, NaH_2_PO4 1.25, MgSO_4_ 10, CaCl_2_ 0.5, NaHCO3 26, Sucrose 234, and Glucose 11, saturated with 95% O2 and 5% CO2) with a Leica VT1200 blade microtome (Leica Microsystemes, Nanterre, France). After cutting, the hippocampus was extracted from the slice and transferred in oxygenated ACSF (in mM: NaCl 119, KCl 2.5, NaH_2_PO_4_ 1.25, MgSO_4_ 1.3, CaCl_2_ 2.5, NaHCO_3_ 26, and Glucose 11) at 37 ± 1 °C for 30 min and then kept at room temperature for at least 1 h before recordings.

#### Electrophysiological recordings

Each slice was individually transferred to a submersion-type recording chamber and continuously superfused (2 ml/min) with oxygenated ACSF at 28 °C. Extracellular recordings were obtained from the apical dendritic layers of the hippocampal CA1 area, using glass micropipettes filled with ACSF. Field excitatory postsynaptic potentials (fEPSP) were evoked by the electrical stimulation of Schaeffer collaterals afferent to CA1. The magnitude of the fEPSPs was determined by measuring their slope. Signals were acquired using a double EPC 10 Amplifier (HEKA Elektronik Dr. Schulze GmbH, Germany), recorded with Patchmaster software (HEKA Elektronik Dr. Schulze GmbH, Germany) and analyzed with Fitmaster software (HEKA Elektronik Dr. Schulze GmbH, Germany).

Input/output (I/O) curves characterizing basal glutamatergic transmission at CA3-CA1 synapses were constructed by plotting mean fEPSP slope against stimulation intensity (10–100 μA).

Facilitation was induced by paired stimulation at different inter-pulse intervals (25–300 ms). Paired pulse ratio (PPR) was calculated by normalizing the slope of the second response to the slope of the first one.

For Long-term potentiation (LTP) experiments test stimuli were delivered once every 15 s and the stimulus intensity was adjusted to produce 40-50% of the maximal response. LTP was induced using a Theta Burst Stimulation (TBS involved 5 trains with 10 bursts of 4 pulses delivered at 100 Hz, an interburst interval of 200 ms and 20 s interval between each train). Average value of fEPSP slope was expressed as a percentage of the baseline response ± SEM.

For Long term depression (LTD) experiments test stimuli were delivered once every 15 s and LTD was induced using a low frequency stimulation protocol (1200 pulses delivered at 0.5 Hz).

### RNA sequencing

HpC tissue from mice stereotactically injected with AAV-shRNA-*Slc20a1*, shRNA-*Slc20a2* or Scramble were collected after cervical dislocation. Total RNA was extracted using TRizol reagent (15596026, ThermoFisher Scientific) and RNeasy Mini Kit (74104, QIAGEN) with DNase I treatment step (79254, QIAGEN) following the manufacturer’s protocol. The integrity of RNA was determined by RNA ScreenTape (5067-5576, Agilent Technologies) on the Agilent 2200 TapeStation (Agilent Technologies). RNA-seq libraries were prepared starting from 100 ng of total RNA using the Ovation mouse RNAseq system (Nugen) as recommended by the manufacturer. Ribosomal RNA was depleted by PCR (10 cycles). RNA-seq libraries were sequenced on an Illumina HiSeq2500 (Paired-End sequencing 130 × 130 bases, High Throughput Mode). A minimum of 10 million paired-end reads was produced per library sample. Sequence reads were aligned to the mouse MM38 reference genome using Hisat2 software and counted by featureCounts from the Subread R package. Only unique reads mapped to known transcripts were used for expression analyses; reads mapped to ribosomal RNA, genome and unmapped reads were excluded.

### In silico analyses

#### Preparing DESeq2 dataclass

The DESeq2 package in R BioConductor (http://www.bioconductor.org/packages/release/bioc/html/DESeq2.html) was used to design the count data into DESeq2 data class. The data is normalized, using log transformation and normalized log transformation and is used for visualization, and differential analysis of high- dimensional count data [98]. The DESeq2 data class consists of a count matrix with rows corresponding to genes and columns denoting experimental samples. Each matrix entry indicates the number of reads that have been unambiguously mapped to a gene in an experimental group.

#### Preprocessing: PCA and quality check

Data entries with reads equal to 0 for more than 75% of each sample (>4/6samples per experimental group) were excluded from further analysis. For dimensional reduction and outlier identification, we performed a principal component analysis (PCA) on the DESeq2 data class of count reads. Euclidean distance was used as a metric for determining orthogonal eigenvectors and eigen values. *prcomp* function in R was used, and variability explained by the eigenvectors were plotted as a biplot representation with the experimental identity (Scramble, shRNA-*Slc20a1*, shRNA-*Slc20a2*). One outlier in shRNA-*Slc20a2* was identified, due to very low read counts, which was subsequently removed. The final biplot and heatmap of the covariance matrix is shown after outlier removal.

#### Differential expression using DESEQ2

The details of the *DESeq2* pipeline is discussed in detail in [98]. Briefly, *DESeq2* package models the data counts on the count matrix using a gamma-Poisson distribution with mean (normalized concentration of cDNA fragments from the gene in a sample). The size factors are determined by the median-of-ratios method. For each gene, a generalized linear model (GLM) is logarithmic fit:

$${\log }_{2}{q}_{{ij}}=\mathop{\sum }\limits_{r}{x}_{{jr}}{\beta }_{{ir}}$$

With *x*_*jr*_ being design matrix elements and *β*_*jr*_ are the coefficients, with *j* belonging to an experimental group relative to a control group for comparison between two groups. The GLM returns overall expression strength of the gene, log_2_ of the fold change (LFC) between the two groups compared (Scramble vs shRNA-*Slc20a1* and Scramble vs shRNA-*Slc20a2*).

Next, variability between replicates is modeled by a dispersion parameter α_*i*_, used to describe the variance of counts as:

$${Var}{K}_{{ij}}={\mu }_{{ij}}+{\alpha }_{i}{\mu }_{{ij}}^{2}$$

Each gene is taken to estimate gene-wise dispersion estimate (maximum likelihood). We determined the dispersion of data counts against the average expression to estimate the distribution of these estimates. An empirical Bayes approach yields an estimate of how close the dispersions fit and the degrees of freedom. An ordinary GLM is performed to obtain maximum-likelihood estimates (MLEs) for the LFCs and then fit a zero-centered normal distribution to the observed distribution of MLEs over all genes. The data counts can be transformed using the regularized logarithmic transformation (*rlog*) by fitting each gene a GLM. The *rlog* transformation accounts for variation in sequencing depth across samples. The other transformation is variance-stabilizing transformation (*vst*) for over-dispersed counts. A maximum a posteriori (MAP) as the final estimate is plotted for the experimental groups (Scramble vs shRNA-*Slc20a1* and Scramble vs shRNA-*Slc20a2*). The genes found in the MAP to be significant in expression at an estimated false discovery rate (FDR) of 10% are depicted in red. The standard outlier after the GLM fit is determined by the Cook’s distance, defined as the scaled distance that the coefficient vector of the GLM would move if the gene in a sample were removed. The model coefficient from the GLM fit for each gene is compared to a level of zero. A Wald test is used to determine the significance of the LFC, the shrunken LFC estimate is divided by the error to yield a z-statistic and compared to a standard Gaussian distribution. The p values thus obtained are adjusted for multiple testing using the Benjamini and Hochberg procedure.

#### Gene network analysis by WGCNA

A major caveat for differential analysis like DESeq2 is that it treats each gene as a separate entity and assumes independence of genes. However, in order to gain an insight into complex biological mechanisms of regulation and co-expression. In order to visualize the changes in genes as modules of a network, we used the WGCNA (Weighted Genes Co-expression Network Analysis) pipeline to analyze gene groups in modules (https://cran.r-project.org/web/packages/WGCNA/index.html) [47]. We selected the top 10% of the most variable genes in the entire dataset across the experiments using a standard variance calculation. For a ($n\times n)$) count matrix, an adjacency matrix is measured as a weighted Pearsons’ correlation:

$${s}_{{ij}}=\frac{1+{corr}({{gene}}_{i},{{gene}}_{j})}{2}$$

Relying on the adjacency matrix, clustering is based on proximity of pairs of genes. WGCNA uses a topological overlap measure (TOM) which replaces the pairwise elements *s*_*ij*_ by:

$${{TOM}}_{{ij}}=\frac{{\sum }_{k\ne i,j}{s}_{{ik}}{s}_{{kj}}+{aij}}{\min ({\sum }_{k\ne i}{s}_{{ik}},{\sum }_{k\ne j}{s}_{{kj}})+1-{s}_{{ij}}}$$

Similarity of two genes *i,j* is determined by the strength of their interaction with the same neighbor genes. To enhance the strength of the correlations, a power transformation is applied: ${a}_{{ij}}={s}_{{ij}}^{\beta },\beta \,>\, 1.$ The power of the system networks is characterized by a power-law distribution, called scale free, with the adjacency equally distributed on [0,1]. We used a soft-power threshold. The connectivity of a network is defined as the total mean of the final adjacency matrix (TOM):

$$CC\left(A\right)=\frac{1}{n(n-1)}\sum _{i}\mathop{\sum }\limits_{j\ne i}{{TOM}}_{{ij}}$$

Modules of genes are constructed by applying hierarchical clustering based on the TOM adjacency matrix. Each module is computed by taking the first principal component and designated as an eigengene. The whole network connectivity distribution is shown as a network heatmap, and the branches in the hierarchical clustering dendrograms correspond to modules. The modules were color-coded and data was segregated from each module to classify them into major clusters/modules. We used the following parameters for our analysis: Minimum module size (minMod) = 25; Power of scale free network (ds) = 3; Dynamic Cut Height = 0.9999; Soft threshold = 0.12.

#### Determination of categories

WGCNA clustering yielded 21 clusters/modules with the experimental groups analyzed. In order to categorize the clusters further, we selected the main trends of the gene expression variability. Using the mean of the cluster Z-scores obtained for each gene in the WGCNA, we selected the modules were determined using an ANOVA test on the cluster z-scores across the experimental conditions: Scramble, shRNA-*Slc20a1* and shRNA-*Slc20a2*. We identified 6 responsive trends and one category with no significant difference. In order to account for the degree of similarity between the components in the categories and cross-sectional similarities, an autocorrelation of the obtained clusters was performed.

#### Gene ontology analysis

Using the six categorized pools of genes that change in particular fashion across the experimental groups, we performed a gene ontology (GO) analysis. GO refers to an enrichment analysis which finds the functional implications of a set of genes that change under certain conditions. The genes are annotated using annotator sets dedicated to the genes. GO leads to a list of significant shared GO terms (or parents of GO terms) used to describe the set of genes, the background frequency, the sample frequency, expected *p*-value, an indication of over/underrepresentation for each term, and *p*-value. The *p*-value is the probability of observing at least a subset of genes out of a total number of genes in the list annotated to a particular GO term. Our GO analysis encompassed the following: Biological processes, Cellular components, Molecular components, KEGG pathway and Reactome pathways. We used the publicly available protocol in Metascape (www.metascape.org/) [99]. Metascape combines functional enrichment, interactome analysis, gene annotation, and membership search to leverage over 40 independent knowledgebases. The minimum overlap was kept at 3, the p value cutoff at 0.01 and the minimum enrichment was kept at 1.5. For the final presentation, the top 20 percent of GO terms were selected. For the mapping of interactome networks, we used String-db (https://string-db.org). The network type was set at full network, network edges defined by confidence of the highest threshold (90%). The specific compartments of enrichment and processes for representation, we selected the top 2 GO term for cellular compartment, biological processes and KEGG pathway descriptors.

### Statistical analysis

All data were analyzed using GraphPad Prism v5 software. For all behavioral experiments, the effect of treatment was analyzed using two-way ANOVAs with repeated measures where appropriate. Significant ANOVAs were also analyzed using Fisher’s PLSD tests where appropriate. All main effects and interactions are noted in the text or figures. Results from data analyses are expressed as means ± SEM. Alpha was set to 0.05 for all analyses. *p < 0.05, **p < 0.01, ***p < 0.001.

### Supplementary information

Supplemental Figure Legends

Figure S1

Figure S2

Figure S3

Figure S4

Figure S5

Figure S6

Original Data File

Reproducibility checklist

## Data Availability

The RNAseq datasets generated and analyzed during the current study are available from the corresponding author upon request.
