# Supplementary material for: Slc20a1 and Slc20a2 regulate neuronal plasticity and cognition independently of their phosphate transport ability
Source: Cell Death Dis. 2024 Jan 9;15(1):20. doi: 10.1038/s41419-023-06292-z (PMC10776841; doi:10.1038/s41419-023-06292-z)
Supplement: Supplementary file 1 — Supplemental Figure Legends [file 41419_2023_6292_MOESM1_ESM.docx]

**Supplemental figure legends**

**Figure S1: PiT-1 and PiT-2 are the most abundant Na-Pi co-transporters in the brain. A**) Relative expression of *Slc17a* (*Slc17a1, Slc17a2 and Slc17a3*) and *Slc34a* (*Slc34a1, Slc34a2, Slc34a3, Slc34a4*) genes in kidney, liver, muscle, and various parts of the brain (Midbrain, Hippocampus (HpC), Cortex, Brainstem (BS), and Cerebellum (CB), n=3 adult mice). **B**) Measurement of the extracellular Pi concentration (mM) in the culture medium of primary HpC neurons after transfection with either pSicoR empty vector (GFP) or shRNA-*Slc20a2*. Culture Medium alone was also measured as a control. **C**) Representative images of Von Kossa staining of primary HpC neurons after lentiviral infections with either shRNA-*Slc20a2* or shRNA-Scramble (control). No formation of calcium-phosphate crystals was observed in this neuronal culture. **D**) Fluorescent microscopy images of a brain cross-section collected 3 weeks after stereotactic injections with control AAV (Scramble). The constructs, expressing GFP, allow to confirm the site and the efficacy of infection. **E**) *Slc20a1* and *Slc20a2* relative expression in the HpC after local stereotactic injections of either Scramble, shRNA-*Slc20a1* or shRNA-*Slc20a2*. **F**) Relative expression of *Slc17a* (*Slc17a1*, *Slc17a2* and *Slc17a3*) and *Slc34a* (*Slc34a1*, *Slc34a2*, *Slc34a3*, *Slc34a4*) in the HpC, 3 weeks after stereotactic injections with AAV9 expressing either shRNA-*Slc20a1*, shRNA-*Slc20a2* or shRNA-Scramble*.* These measurements were obtained from n= 5 animals for *shRNA-Scramble* and shRNA-*Slc20a1,* and n=4 for shRNA-*Slc20a2*. Quantification of mRNA expression is relative to the *shRNA-Scramble* group. **G**) Preference index of the right and left location of the objects used in the NOR paradigm. Mice tested were injected with either Scramble, shRNA-*Slc20a1* or shRNA-*Slc20a2*. No initial preference for any exposed object (A or B) in any orientation (right/left) was observed in any group. **H**) Open field test (OFT) and **I**) Dark and Light transition test (D/LT) performed 3 weeks after stereotactic injections with either Scramble, shRNA-*Slc20a1* or shRNA-*Slc20a2* (n=27/28 mice per group, from 3 independent cohorts). Entries and time spent in lit compartment were evaluated.

All behavioral tests were performed on at least two independent experiments (for each experiment: n≥10-8 mice per group). Data are expressed as mean ± s.e.m. *p ≤ 0.05, **p ≤ 0.01, ***p ≤ 0.001, NS: not significant, by two-tailed Student’s t test or 2-way ANOVA followed by Tukey’s multiple comparisons test compared to the control group.

**Figure S2: Hierarchical clustering obtained from differentially expressed genes after hippocampal downregulation of either *Slc20a1* or *Slc20a2*.** **A-J**) RNAseq analysis performed on HpC samples after downregulation of either *Slc20a1* or *Slc20a2*. **A**) Scatterplot showing the dispersion of the genes against the mean of normalized counts, the estimation of the final distribution for the differentially expressed genes. **B**) Hierarchical clustering obtained from differentially expressed genes in (i) Scramble vs shRNA-*Slc20a1* and (ii) Scramble vs shRNA-*Slc20a2*. The clustering is obtained from the analytical pipeline DESeq2. **C**) Topographical overlay map (TOM) plot obtained from clustering the genes. Rows and columns

correspond to the modules identified in the dendrogram, and progressive lighter colors indicate

higher topographical overlap. Modules with high connectivity are located at the tip of the module branches indicating higher interconnectedness. **D**) Heatmap of the autocorrelation matrix showing 2 the identical expression of variability of each category of genes across the groups. Autocorrelation matrix scale: -0.75 to 0.75. **E**) Categorization of the genes described in Fig. 2. (I) and the identified clusters in each category. The heatmap represents the mean cluster z-score of the genes in the corresponding cluster. Scale- 0.5 to 1. **F**) Mean z-score of each group in corresponding clusters. Data is represented as Mean ± SD. **G**) Gene ontology network obtained from genes in Cluster 2 showing enrichment for synaptic plasticity and its associated functions. **H**) Gene ontology network obtained from genes in Cluster 3 showing enrichment for synaptic plasticity and its associated functions. **I**) Gene ontology network obtained from genes in Cluster 4 showing enrichment for synaptic plasticity and its associated functions. **J**) Gene ontology network obtained from genes in Cluster 6 showing enrichment for synaptic plasticity and its associated functions.

**Figure S3: PiT-2 is essential to maintain neuronal branching in the hippocampus.** **A**) Analysis of neuronal branching in primary HpC neurons transfected at DIV11 with either pSicoR empty vector (GFP) or shRNA-*Slc20a2*. GFP was used to identify the transfected neurons and visualize/analyze neuronal branching. Total number and length of neurites (μm), and Sholl analysis were assessed at 24, 48, 72 and 96 hours post-transfection (n=12 neurons per group). **B**) Representative images of immunofluorescence for PiT-1 or PiT2 performed in primary HpC neuron co-transfected with pSicoR plasmid (EGFP) and plasmid expressing either human WT SLC20A1, WT SLC20A2, Pi transport-deficient mutant SLC20A1 (SLC20A1-S128A) or Pi transport-deficient mutant SLC20A2 (SLC20A2-S113A). These images validate the induction of an ectopic expression of *SLC20A1* or *SLC20A2* WT and mutated forms in primary HpC neurons. pSicoR plasmid expresses GFP which allows us to identify the transfected primary HpC neurons in the cultures. **C**) Western blot performed in HEK293 cells after transfection with plasmid expressing either human WT SLC20A1, WT SLC20A2, Pi transport-deficient mutant SLC20A1 (S128A), Pi transport-deficient mutant SLC20A2 (S113A) or control. PIT2 was only detected in the cells transfected with WT SLC20A2 or Pi transport-deficient mutant SLC20A2 (S113A). PIT1 was only detected in the cells transfected with WT SLC20A1 or Pi transport-deficient mutant SLC20A1 (S128A). **D**) Total number of neurites and neurite length (μm) measurements performed on primary HpC neurons transfected with either WT SLC20A2, Pi transport-deficient mutant SLC20A2 (SLC20A2-S113A) or control. These measurements were obtained from n=20-47 individual neurons from 3 independent neuronal preparations. **E**) In situ hybridization (RNAscope) of *Slc20a2* (green) mRNA and either *Gfap (astrocyte marker)* or *Iba1* (microglial cell marker) (in red) transcript on hippocampal brain cross-section (Scale bar = 200 μm). A focus is made on the Dentate Gyrus (DG) areas. The arrows point out colocalization between either *Slc20a2* and *Gfap* or *Slc20a2* and *Iba1* transcripts.

Data are expressed as mean ± s.e.m. *p ≤ 0.05, **p ≤ 0.01, ***p ≤ 0.001, NS: not significant, by two-tailed Student’s t test or 2-way ANOVA followed by Tukey’s multiple comparisons test compared to the control group.

**Figure S4: PiT-1 modulates GABAergic system in the hippocampus.** **A**) Dendritic spine density (number of spines per μm) in primary HpC neurons transfected with either control pSicoR plasmid (EGFP), WT SLC20A1 or Pi transport-deficient mutant SLC20A1 (SLC20A1-S128A). This analysis was performed on n= 22-32 individual HpC neurons per group, from 3 independent neuronal preparations. **B**) Western Blot quantification for total and phosphorylated GluA1 (Ser845 and 831) and GABAR2 in HpC injected with either Scramble or shRNA-*Slc20a1*. β-actin was used as a loading control for each sample. **C**) Functional enrichment analysis of GABAergic and membrane trafficking related genes altered in the HpC after *Slc20a1* downregulation.

Results are given as mean ± s.e.m. *p ≤ 0.05, **p ≤ 0.01, ***p ≤ 0.001 by Student’s t test or oneway ANOVA and Tukey’s post hoc compared to control group.

**Figure S5: Hippocampal downregulation of *Slc20a1* affects membrane trafficking.** **A**) Relative expression of Gad65 and Gad67 in the HpC after stereotactic injections of either shRNA-*Slc20a1* or Scramble. These measurements were obtained from n=8 mice. **B**) Western Blot quantification for Otof, Syt2 and Rab5 in HpC injected with either AAV-shRNA-*Slc20a1* or AAV shRNA-Scramble. β-actin was used as a loading control for each sample. **C**) GABAR2 immunofluorescence (scale bar = 50μm) performed on brain cross-sections at the level of the DG after Slc20a1 silencing and compared to controls. Brains were collected from two independent cohorts of mice for each group.

Results are given as mean ± s.e.m. *p ≤ 0.05, **p ≤ 0.01, ***p ≤ 0.001, by two-tailed Student’s t test compared to the control group.

**Figure S6: Downregulation of *Slc20a1* leads to Otoferlin overexpression in the hippocampus. A**) Western Blot quantification for Syt2 and Rab5 in the HpC of hippocampi locally injected with either shRNA-*Scramble*, shRNA-*Slc20a1*, shRNA-*Otof,* or shRNA-*Slc20a1* + shRNA-*Otof*. **B**) Preference index during the testing phase (Left panel, exploration time for the novel object/Total exploration) of NOR performed on 3-month-old mice after local stereotactic injections with either: shRNA-*Scramble*, shRNA-*Slc20a1*, shRNA-Otof, or shRNA-*Slc20a1* + shRNA-*Otof* (n=10 mice per group). **C**) As a control, the preference index for the (right versus left) object location or for the object A versus B during the training phase of the NOR (right panel) were measured for each group. We confirm that no initial preference for any exposed object (A or B) or any orientation (right/left) was observed in any groups. The NOR was performed in two independent cohorts. **D**) Western blot quantification for the total and phosphorylated forms of GluA1 (Ser845 and 831), GABAR2, Otof, Syt2 and Rab5 in HpC injected with either shRNA-*Scramble*, shRNA-*Slc20a1*, shRNA-*Otof*, or shRNA *Slc20a1* + shRNA-*Otof.* GAPDH was used as a loading control for each sample.

Results are given as mean ± s.e.m. *p ≤ 0.05, **p ≤ 0.01, ***p ≤ 0.001 by Student’s t test or oneway ANOVA and Tukey’s post hoc compared to control group.
