# Supplementary figures and images for: Slc20a1 and Slc20a2 regulate neuronal plasticity and cognition independently of their phosphate transport ability
Source: Cell Death Dis. 2024 Jan 9;15(1):20. doi: 10.1038/s41419-023-06292-z (PMC10776841; doi:10.1038/s41419-023-06292-z)

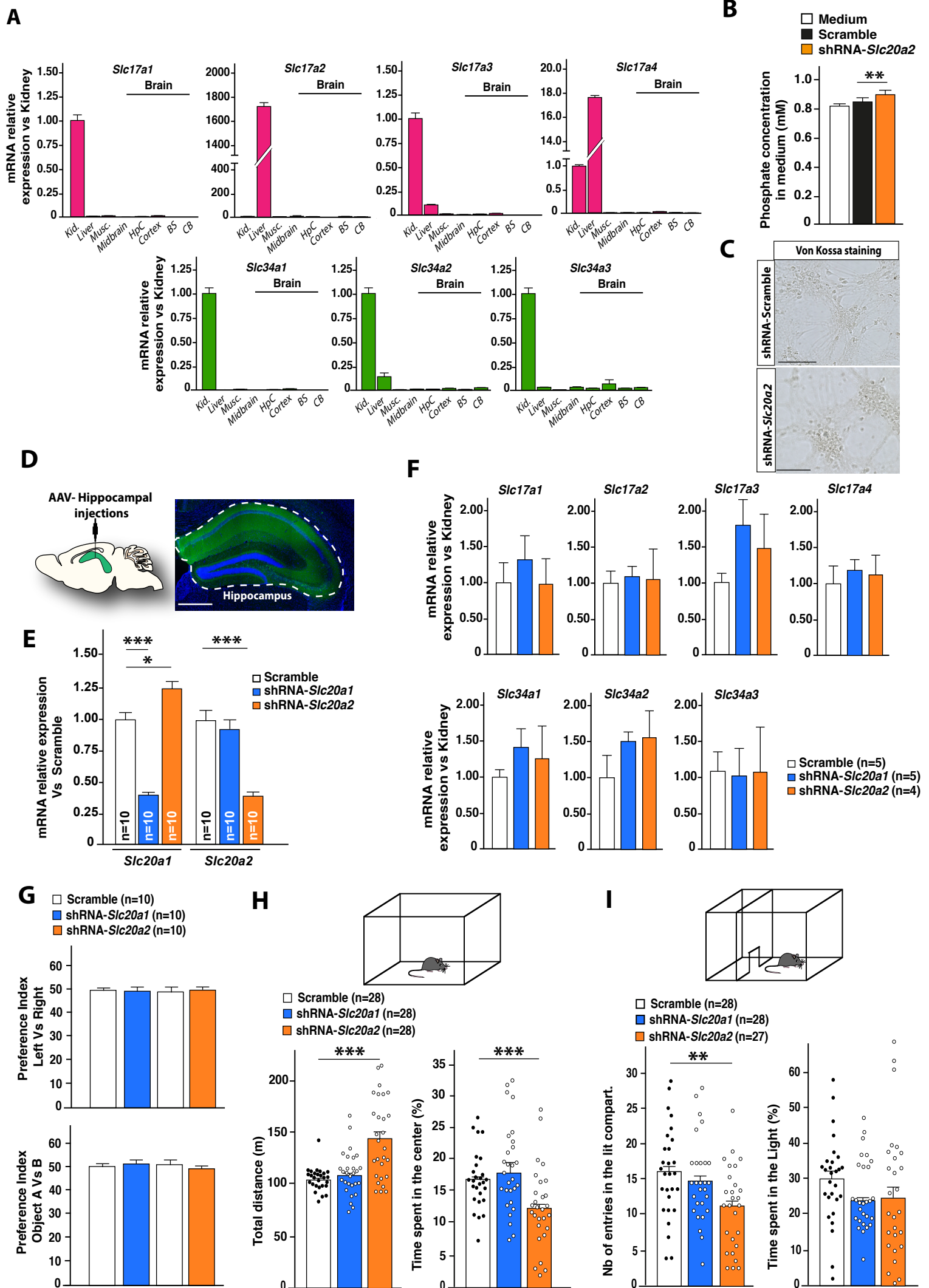

**Figure S1**

Supplement: Supplementary file 2 — Figure S1 [file 41419_2023_6292_MOESM2_ESM.pdf]

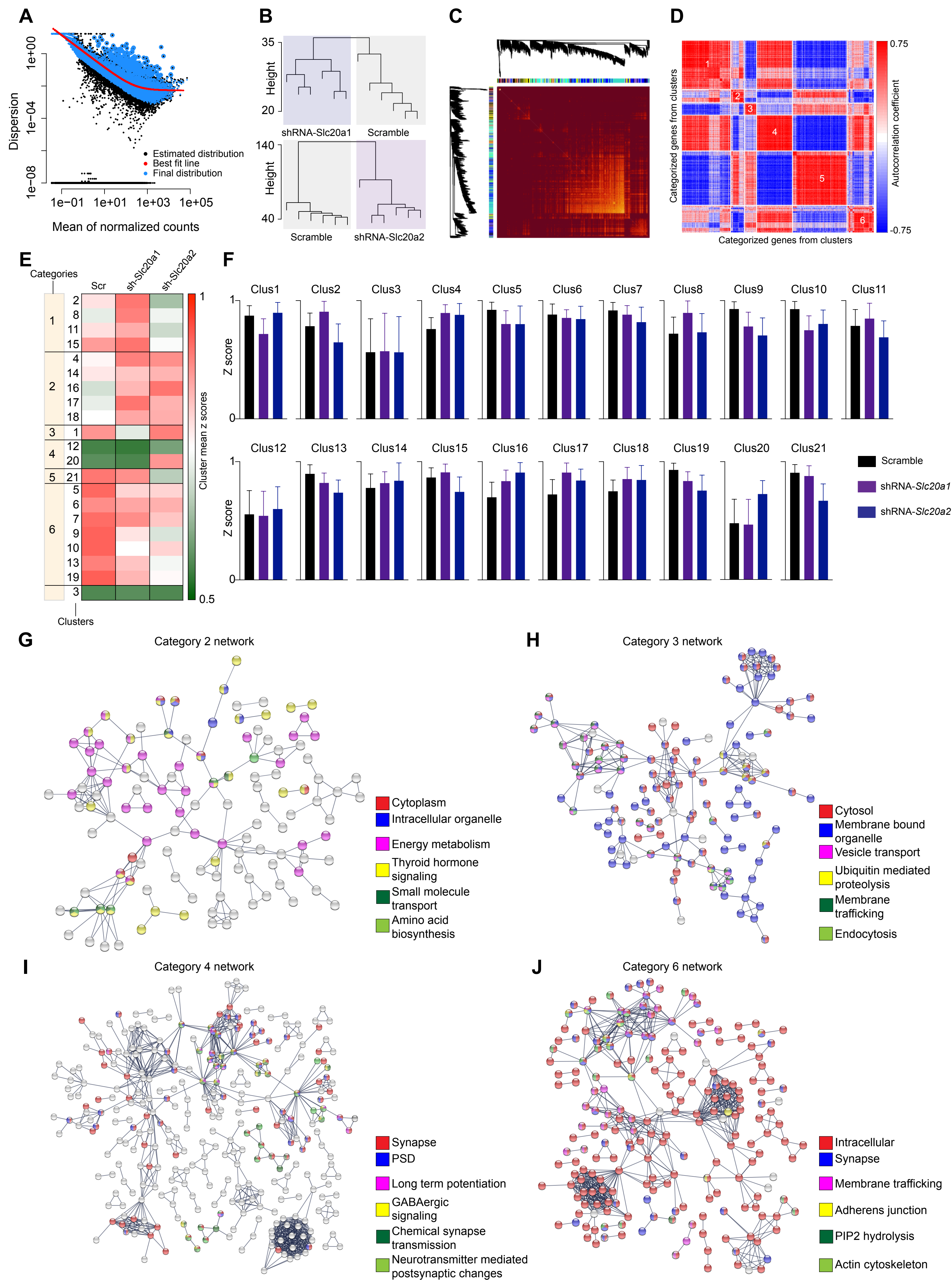

**Figure S2**

Supplement: Supplementary file 3 — Figure S2 [file 41419_2023_6292_MOESM3_ESM.pdf]

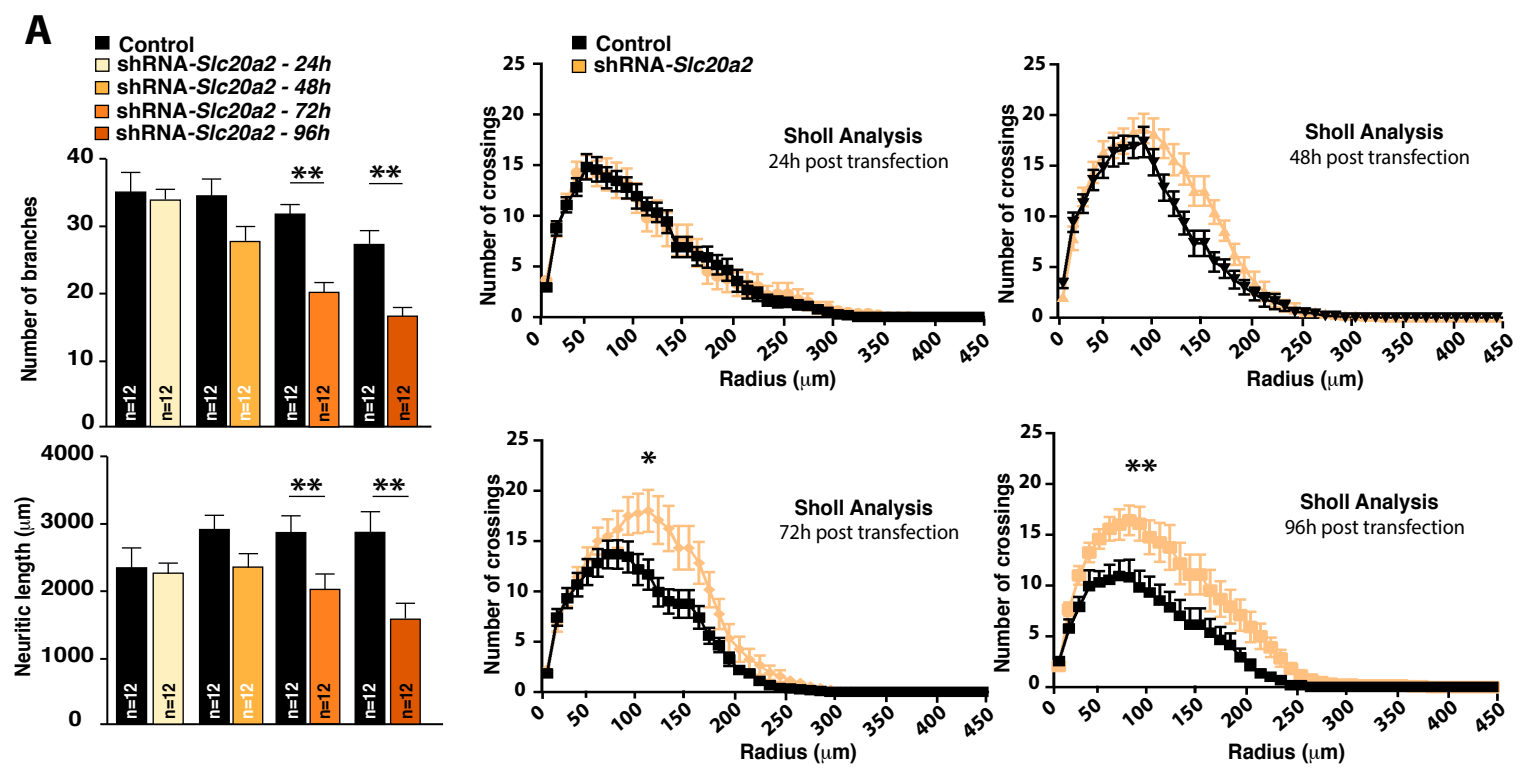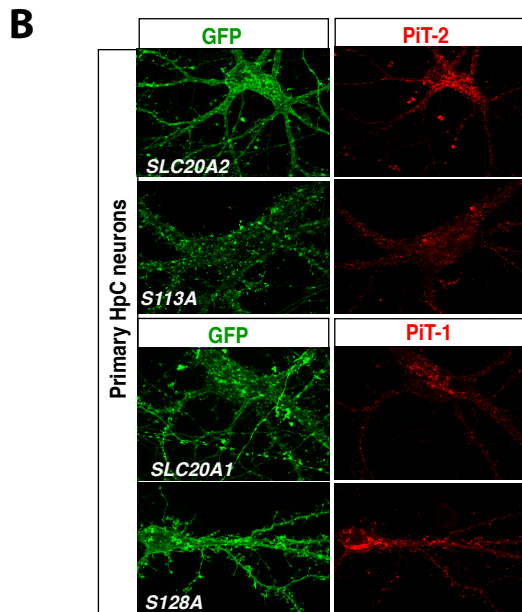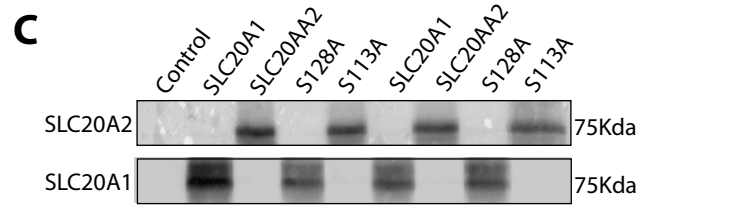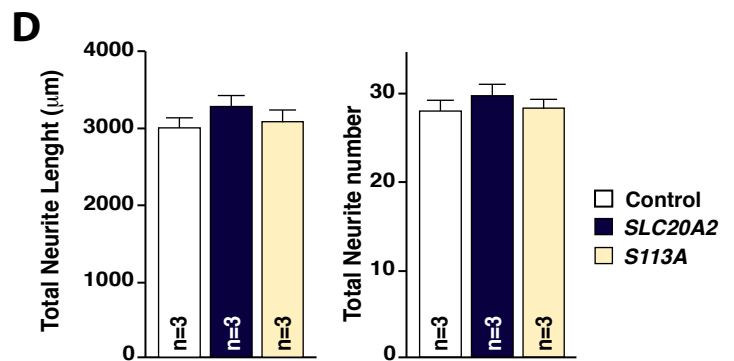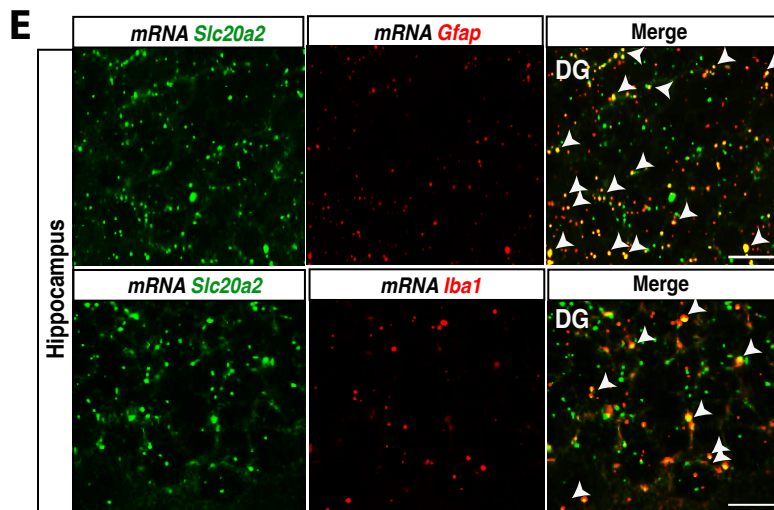

**Figure S3**

Supplement: Supplementary file 4 — Figure S3 [file 41419_2023_6292_MOESM4_ESM.pdf]

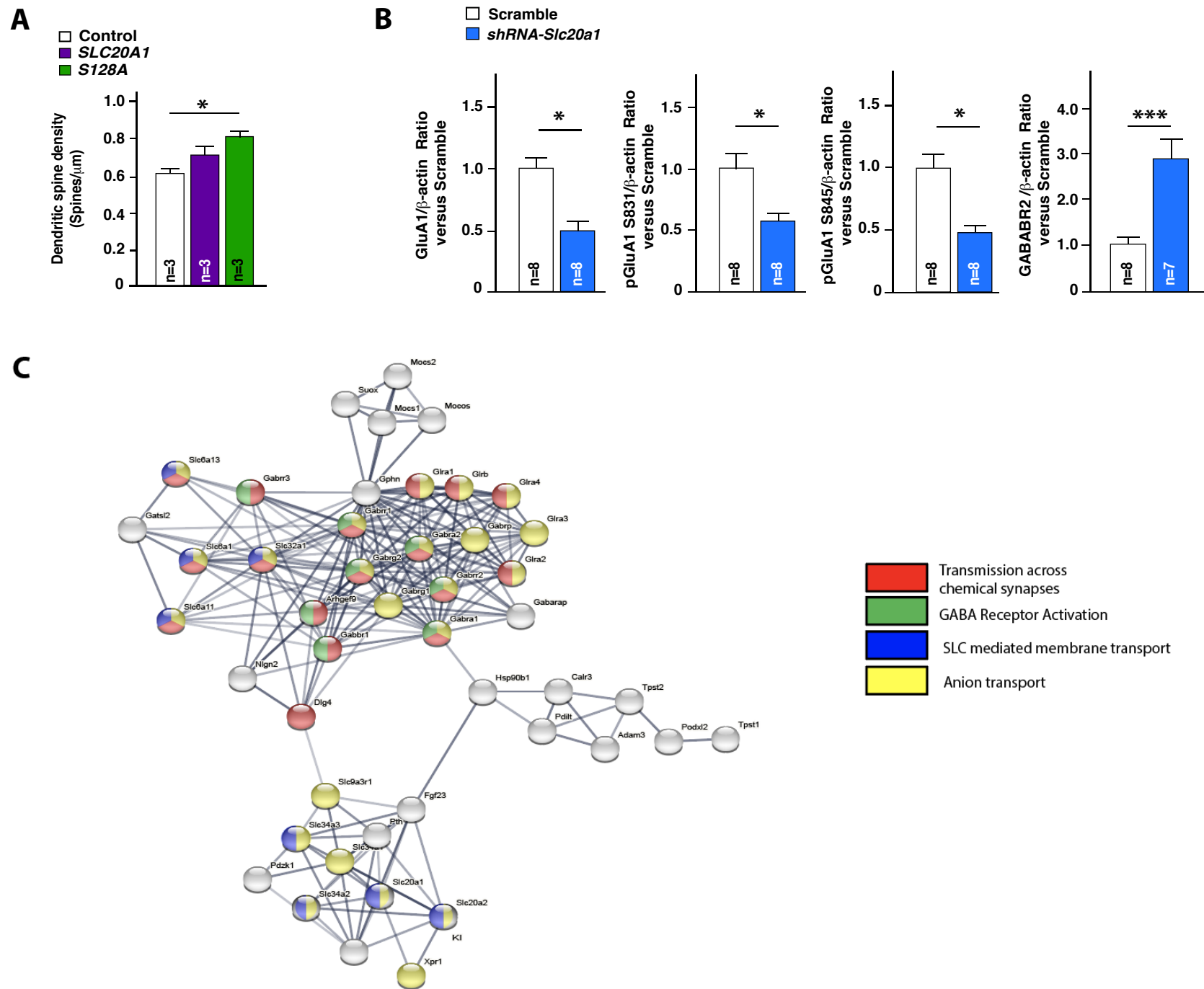

**Figure S4**

Supplement: Supplementary file 5 — Figure S4 [file 41419_2023_6292_MOESM5_ESM.pdf]

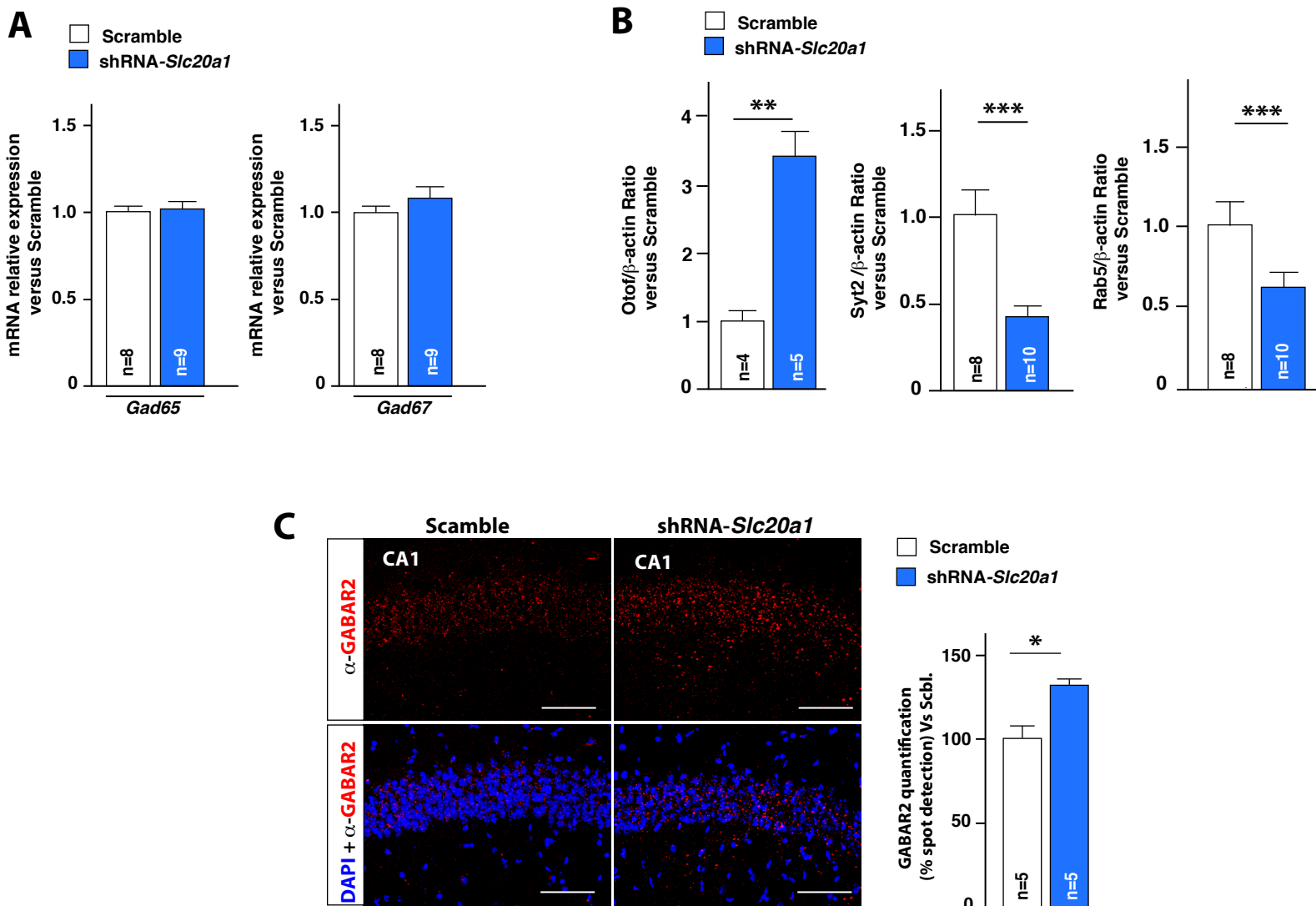

Figure S5

Supplement: Supplementary file 6 — Figure S5 [file 41419_2023_6292_MOESM6_ESM.pdf]

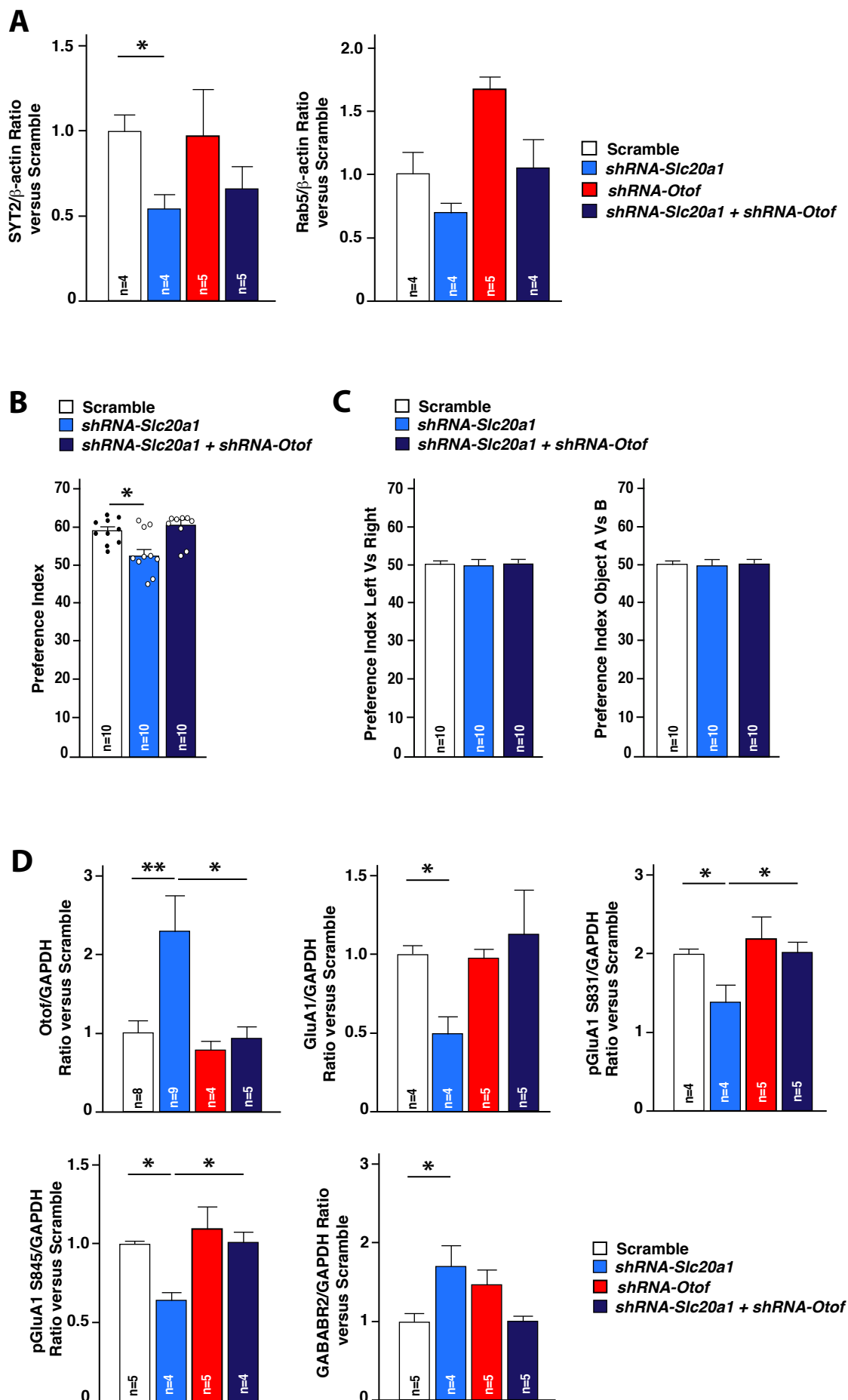

**Figure S6**

Supplement: Supplementary file 7 — Figure S6 [file 41419_2023_6292_MOESM7_ESM.pdf]

Figure S3C

SLC20A2

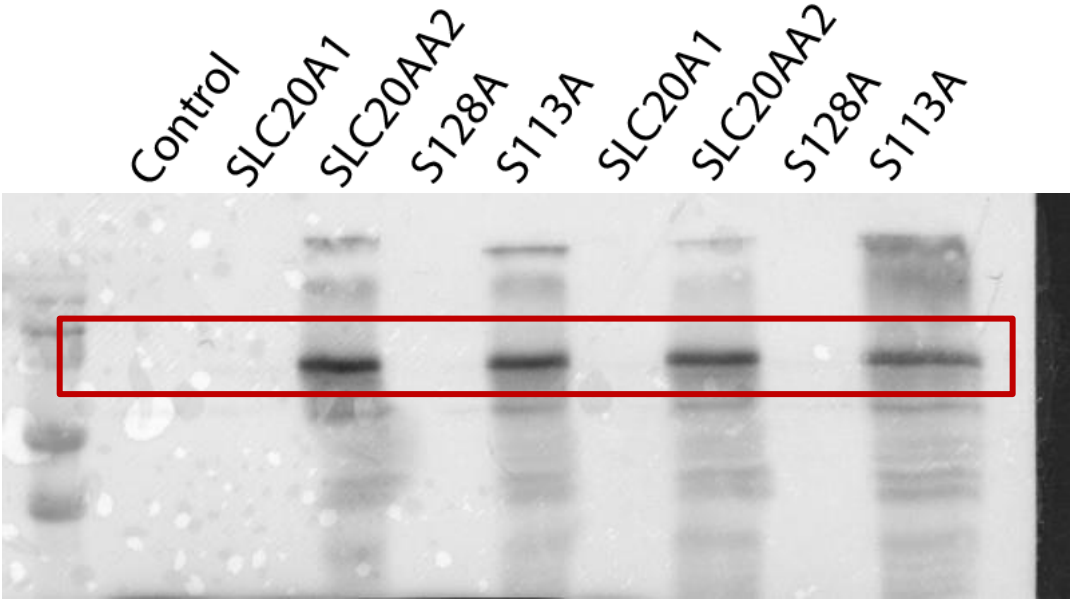

SLC20A1

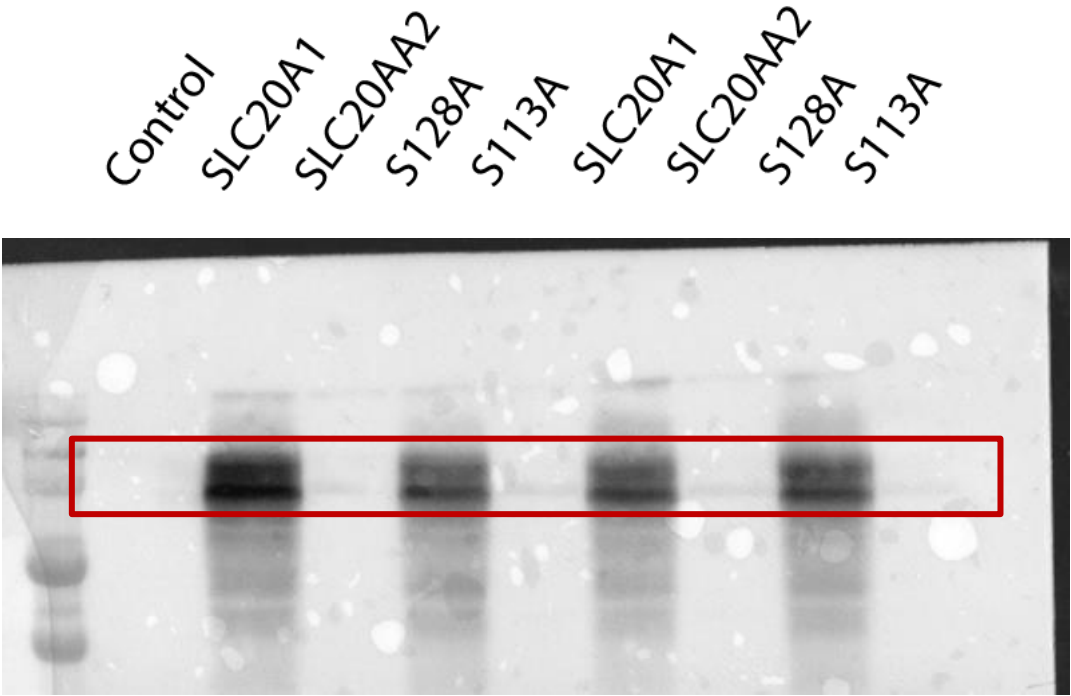

Figure 4B

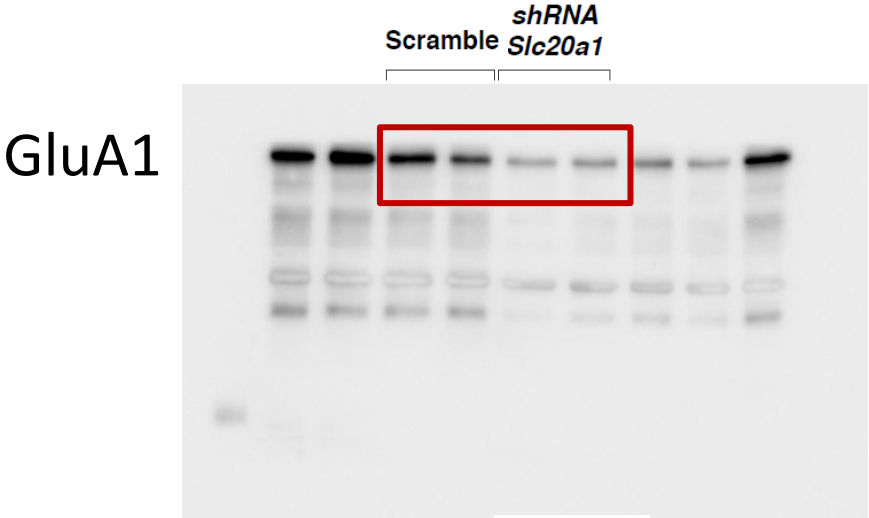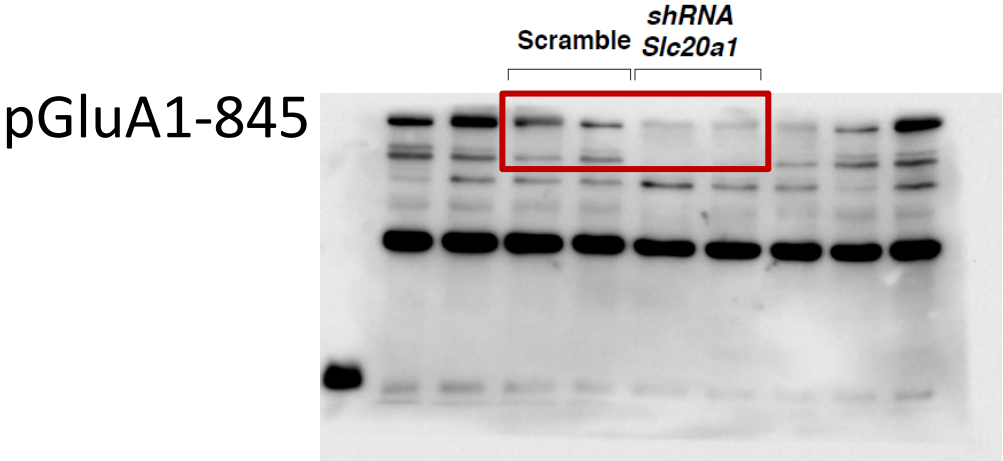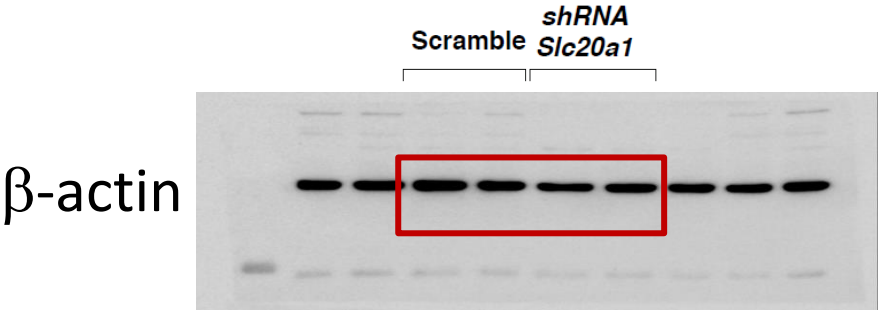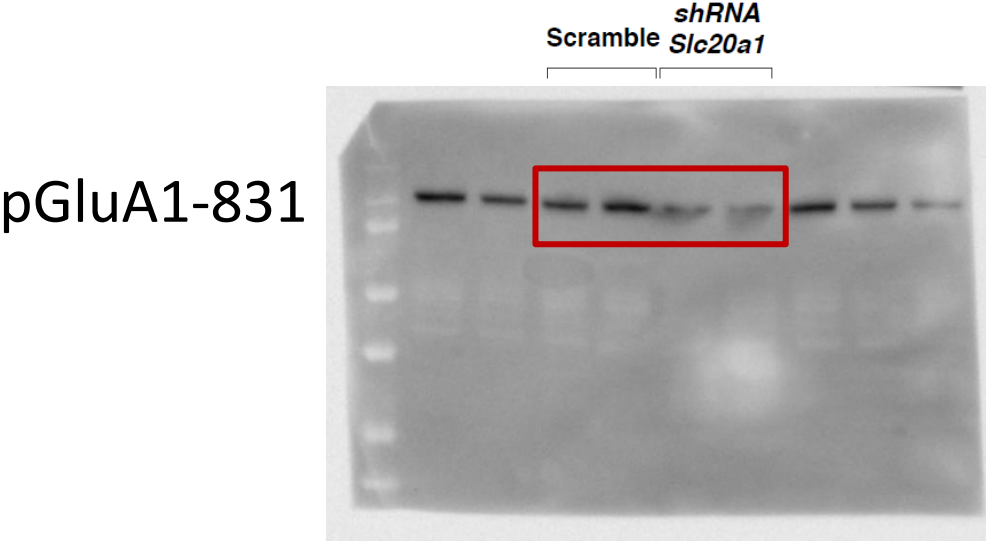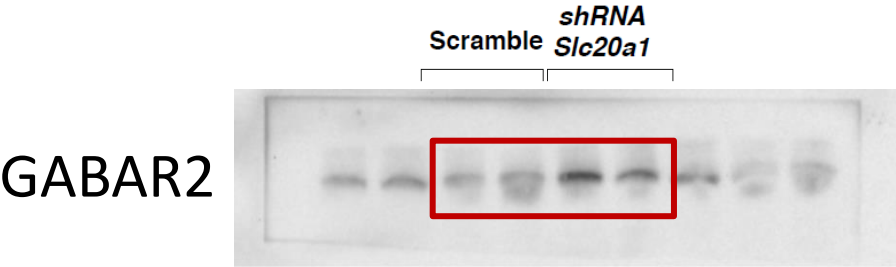

Figure 5B

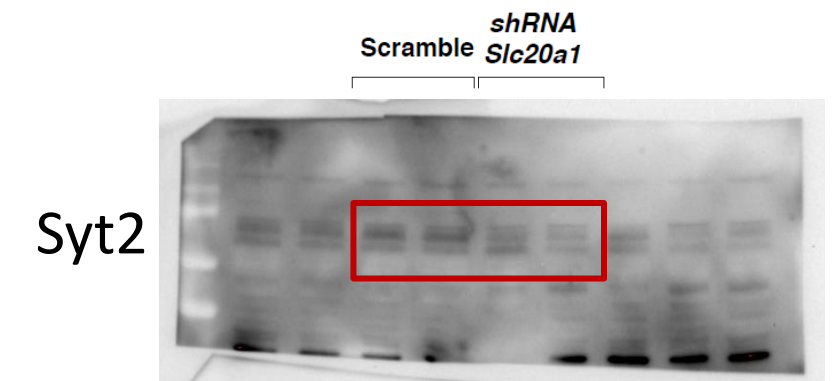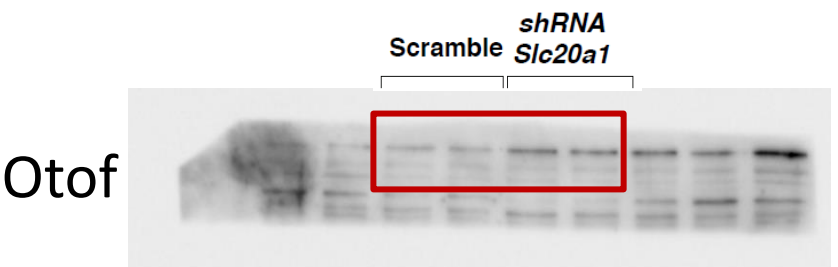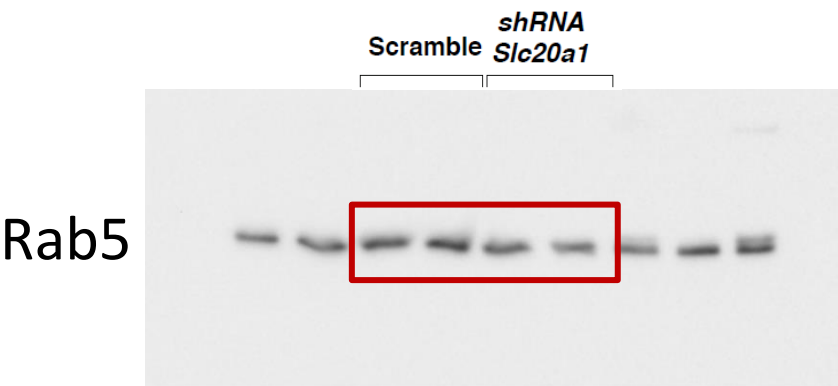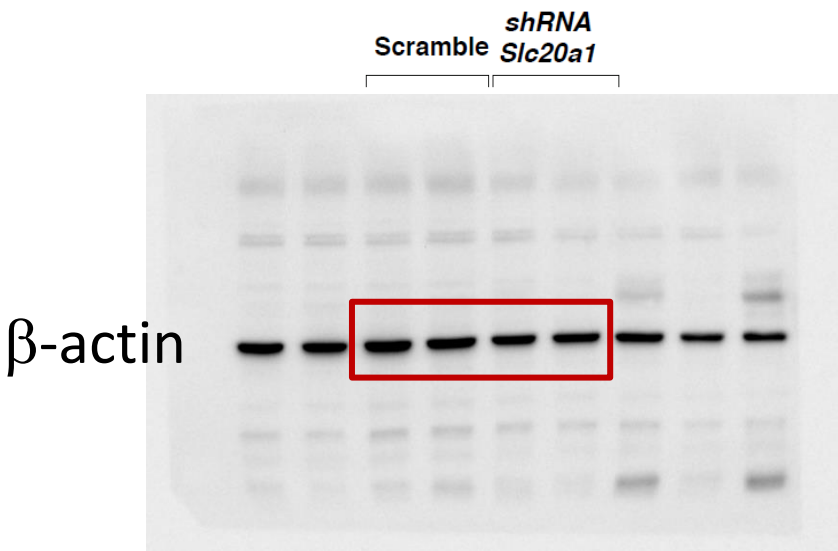

Figure 6B

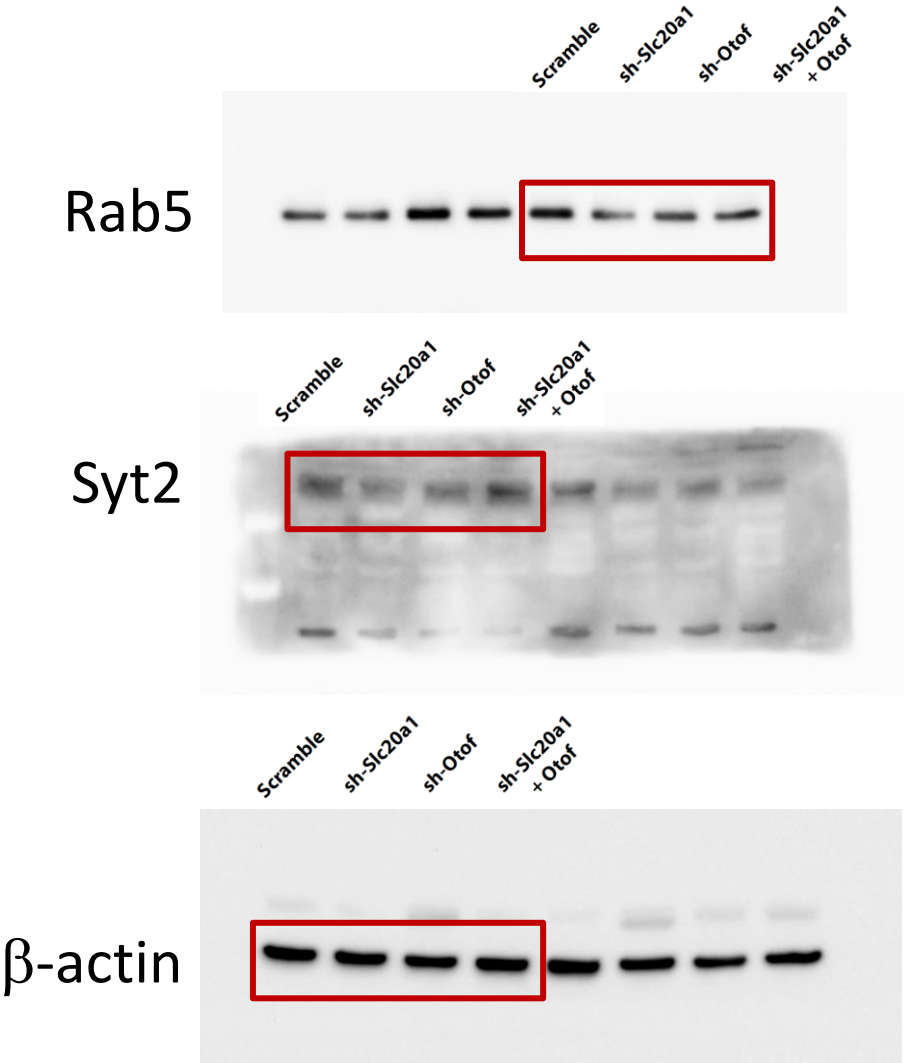

Figure 6G

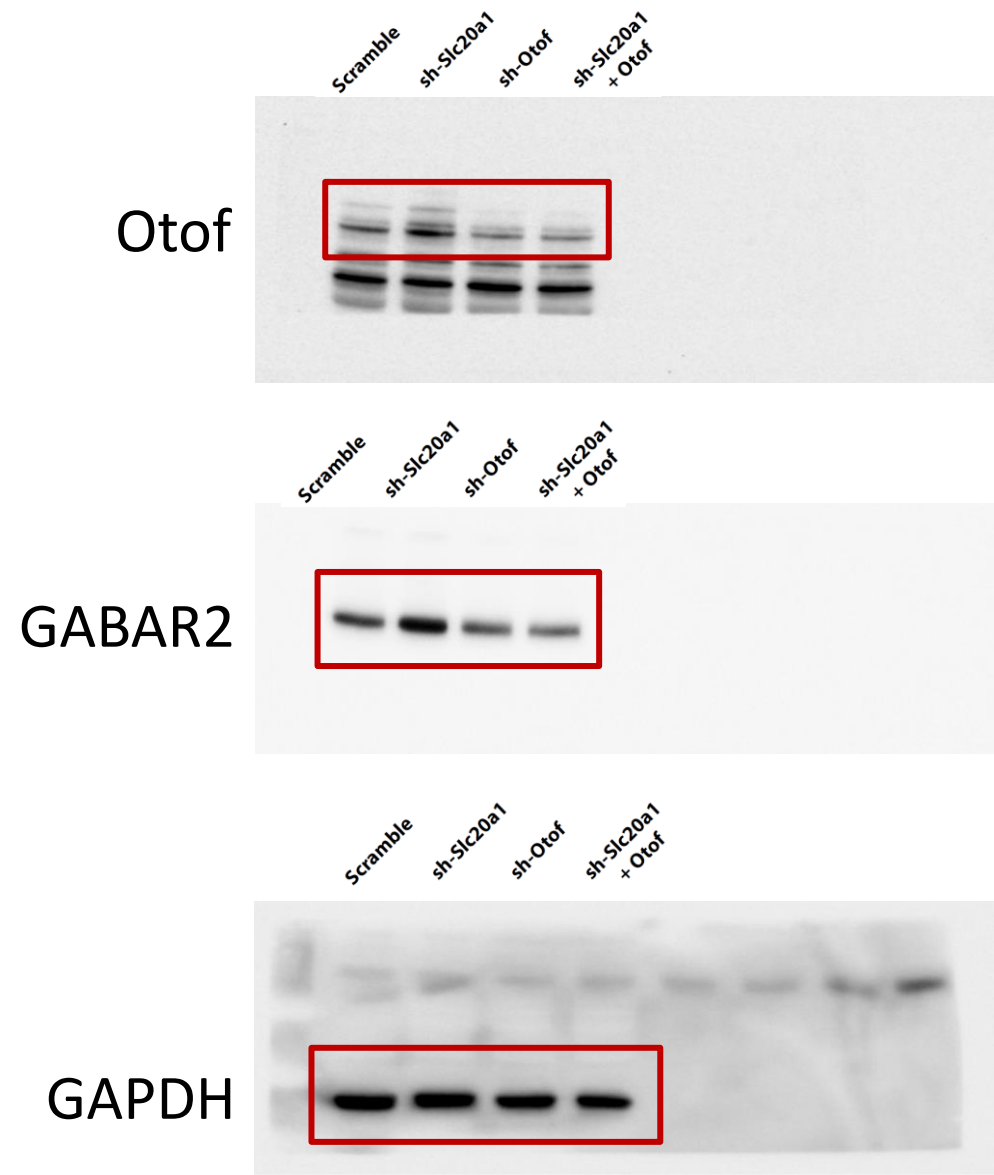

pGluA1-845

pGluA1-831

GluA1

GAPDH

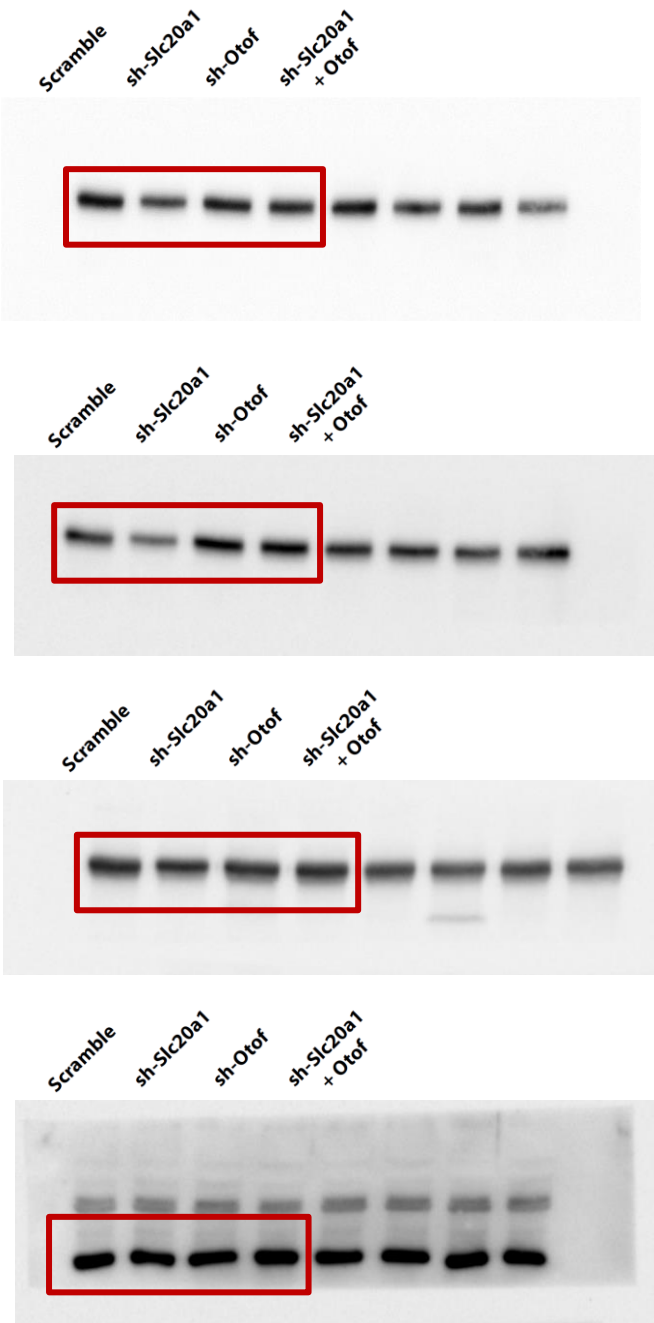

Supplement: Supplementary file 8 — Original Data File [file 41419_2023_6292_MOESM8_ESM.pdf]
